# Long-term efficacy and safety of cardiac genome editing for catecholaminergic polymorphic ventricular tachycardia

**DOI:** 10.20517/jca.2023.42

**Published:** 2024-01-04

**Authors:** Oliver M. Moore, Yuriana Aguilar-Sanchez, Satadru K. Lahiri, Mohit M. Hulsurkar, J. Alberto Navarro-Garcia, Tarah A. Word, Joshua A. Keefe, Dean Barazi, Elda M. Munivez, Charles T. Moore, Vaidya Parthasarathy, Jaysón Davidson, William R. Lagor, So Hyun Park, Gang Bao, Christina Y. Miyake, Xander H. T. Wehrens

**Affiliations:** 1 Cardiovascular Research Institute, Baylor College of Medicine, Houston, TX 77030, USA.; 2 Department of Integrative Physiology, Baylor College of Medicine, Houston, TX 77030, USA.; 3 Department of Neuroscience, Baylor College of Medicine, Houston, TX 77030, USA.; 4 Department of Bioengineering, Rice University, Houston, TX 77030, USA.; 5 Department of Pediatrics (Cardiology), Baylor College of Medicine, Houston, TX 77030, USA.; 6 Department of Medicine (Cardiology), Baylor College of Medicine, Houston, TX 77030, USA.; 7 Center for Space Medicine, Baylor College of Medicine, Houston, TX 77030, USA.

**Keywords:** Catecholaminergic polymorphic ventricular tachycardia, ryanodine receptor, CRISPR/Cas9, RyR2, genome editing

## Abstract

**Introduction:**

Heterozygous autosomal-dominant single nucleotide variants in *RYR2* account for 60% of cases of catecholaminergic polymorphic ventricular tachycardia (CPVT), an inherited arrhythmia disorder associated with high mortality rates. CRISPR/Cas9-mediated genome editing is a promising therapeutic approach that can permanently cure the disease by removing the mutant *RYR2* allele. However, the safety and long-term efficacy of this strategy have not been established in a relevant disease model.

**Aim:**

The purpose of this study was to assess whether adeno-associated virus type-9 (AAV9)-mediated somatic genome editing could prevent ventricular arrhythmias by removal of the mutant allele in mice that are heterozygous for *Ryr2* variant p.Arg176Gln (R176Q/+).

**Methods and Results:**

Guide RNA and SaCas9 were delivered using AAV9 vectors injected subcutaneously in 10-day-old mice. At 6 weeks after injection, R176Q/+ mice had a 100% reduction in ventricular arrhythmias compared to controls. When aged to 12 months, injected R176Q/+ mice maintained a 100% reduction in arrhythmia induction. Deep RNA sequencing revealed the formation of insertions/deletions at the target site with minimal off-target editing on the wild-type allele. Consequently, CRISPR/SaCas9 editing resulted in a 45% reduction of total *Ryr2* mRNA and a 38% reduction in RyR2 protein. Genome editing was well tolerated based on serial echocardiography, revealing unaltered cardiac function and structure up to 12 months after AAV9 injection.

**Conclusion:**

Taken together, AAV9-mediated CRISPR/Cas9 genome editing could efficiently disrupt the mutant *Ryr2* allele, preventing lethal arrhythmias while preserving normal cardiac function in the R176Q/+ mouse model of CPVT.

## Graphical Abstract

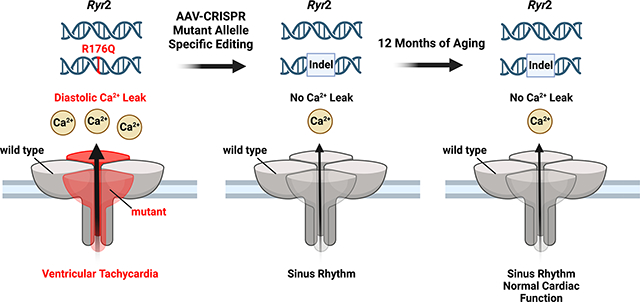

## INTRODUCTION

Catecholaminergic polymorphic ventricular tachycardia (CPVT) is an inheritable disorder associated with polymorphic or bidirectional ventricular tachycardia triggered by emotional or physical stress without underlying cardiac structural abnormalities. CPVT is most commonly diagnosed in children or young adults^[1]^. Although many CPVT patients are treated with beta-adrenoceptor blockers, up to 30% still encounter a cardiac event within 8 years following their initial diagnosis^[2]^. Unlike other disorders associated with ventricular arrhythmias, implantable cardioverter-defibrillators (ICDs) might be ineffective in a subset of patients with CPVT such as those with incessant ventricular arrhythmias^[3,4]^. Moreover, death can occur despite ICD implantation. While left cardiac sympathetic denervation may lead to a reduction in symptoms for patients who are refractory to medication, lethal arrhythmias can still occur^[5]^. In addition, these medical and surgical approaches are all associated with adverse side effects, including bradycardia, hypotension, fatigue, and Horner’s syndrome^[6]^. In addition, noncompliance and missed doses of antiarrhythmic medications can potentially lead to lethal events^[1,3]^. Thus, the demand for more potent and efficacious treatments for CPVT that address the underlying genetic causes remains high.

CPVT is most commonly caused by autosomal-dominant variants in the *RYR2* gene encoding the type 2 ryanodine receptor (RyR2)^[7]^. These CPVT causative RyR2 variants tend to cluster in N-terminal, central, and C-terminal domains^[8]^. Despite being located within different parts of the channel structure, the CPVT variants all increase sarcoplasmic reticulum (SR) calcium (Ca^2+^) leak during diastole, which can initiate delayed afterdepolarizations and promote triggered activity-induced ventricular arrhythmias^[9,10]^. CPVT variants in *RYR2* can also predispose to atrial arrhythmias in a subset of patients^[11]^.

We have previously shown that genome editing of the mutant *Ryr2* allele could correct the CPVT phenotype and lethal arrhythmias in a p.Arg176Gln (R176Q/+) heterozygous mouse model^[12]^. Specifically, “clustered regularly-interspaced short palindromic repeats” (CRISPR) paired with Cas9 endonucleases were shown to alter the genome by introducing double-stranded breaks (DSBs) at a specific DNA sequence targeted using a guide RNA (gRNA)^[13]^. Our prior study revealed that repair of these DSBs by non-homologous end joint (NHEJ) introduced insertions and deletions (indels) into the mutant *Ryr2* allele, thereby reducing the *Ryr2* mRNA and RyR2 protein levels. Moreover, SR Ca^2+^ handling was normalized within ventricular myocytes isolated from R176Q/+ heterozygous mice. However, one caveat was that these studies did not establish the safety and long-term efficacy of this CRISPR/Cas9 approach. Moreover, the gRNA used in the prior study targeted a silent restriction site present exclusively in the mutant R176Q/+ allele upstream of the actual genetic variant. Therefore, to determine if the disease-causing mutation site could be targeted directly, we designed a different gRNA. Our present studies reveal that the CRISPR/Cas9 genome editing system directly targeting the actual mutation site *Ryr2* GT527–528AA can specifically and efficiently edit the mutant allele *in vivo*. Characterization of the R176Q/+ mice post-genome editing revealed the absence of cardiac arrhythmias and stable cardiac function for up to 12 months post-injection. These studies provide proof-of-concept for the long-term effectiveness and safety of cardiac genome editing as a permanent therapy for CPVT in a relevant small animal model.

## MATERIAL AND METHODS

The data supporting the findings of this study are available upon reasonable request from the corresponding author.

### Animal studies

Studies were performed following protocols approved by Baylor College of Medicine’s IACUC, compliant with the Guide for the Care and Use of Laboratory Animals published by the US National Institutes of Health (2011). We used R176Q/+ mice backcrossed into a C57BL/6J background for > 10 generations. The mice underwent genotyping through PCR amplification and restriction digest of an upstream BbsI restriction site^[10,11]^. Pups were randomized at injection; similar numbers of male and female mice were used. Runt mice with a body weight < 2 standard deviations below the litter average were excluded from studies. Experimenters were blinded during studies and analysis.

### Adeno-associated virus

CRISPR *Staphylococcus aureus* Cas9 (SaCas9) guide RNAs were developed to target the variant site in the *Ryr2* gene. Plasmid sequences for SaCas9 [Supplementary Figure 1], cloning details, and methods for the production of AAV9 are described in the Supplementary Materials. Neonatal mice (P10) were subcutaneously injected with 1 × 10^11^ vector genome copies/gram, as described^[12]^. Mice were randomized to control or experimental gRNA at injection.

### Next-generation deep sequencing

Genomic DNA was isolated from bulk ventricular tissues using TRIzol (#15596, Life Technologies, Carlsbad, CA). *Ryr2* gDNA was amplified with primers targeting sequences upstream and downstream of the variant site by Phusion High-Fidelity DNA Polymerase (#M0530S, New England Biolabs, Ipswich, MA). Please refer to the Supplementary Materials for sequencing methods and analysis procedures.

### Quantitative real-time polymerase chain reaction

Total RNA was isolated from ventricular tissue samples using TRIzol (#15596, Life Technologies, Carlsbad, CA) and was reverse transcribed by iScript (#1708841, Bio-Rad; Hercules, CA). Please refer to the Supplementary Materials for primers and details.

### Western blotting

Ventricular tissue was flash frozen in liquid N2 before performing western blots; see the Supplementary Materials.

### Programmed electrical stimulation

Electrophysiology studies were performed in R176Q/+ mice and wild-type littermates at 6 weeks or 12 months post AAV9 injection, as described^[12]^. Following right heart catheterization, mice underwent baseline ECG and intracardiac electrogram recordings, isoproterenol (2 mg/kg) and caffeine (120 mg/kg) injection, and ventricular pacing protocols [Supplementary Materials]. Sustained ventricular arrhythmia was defined as greater than 10 consecutive ventricular beats at a rate faster than 600 bpm, bigeminy, or bidirectional ventricular tachycardia characteristic of CPVT.

### Calcium imaging studies

Mouse hearts were excised, cannulated, and perfused retrogradely using a heated Langendorff system via the aorta, as described^[14]^. Cardiomyocytes were paced at 1-Hz for 15 s, left unstimulated for 60 s, and then perfused with 10 mM caffeine to assess SR Ca^2+^ load. For details, please refer to the Supplementary Materials.

### Echocardiography

Echocardiography studies were performed at 4-, 8-, and 12-months post-AAV9 injection in anesthetized mice [Supplementary Materials].

### Statistical analysis

Results are shown as mean ± standard error of the mean (SEM). The D’Agostino-Pearson test was performed to confirm normality for datasets containing continuous variables before evaluation with the student’s *t*-test or Mann-Whitney’s test for non-parametric data. For categorical variables, the Fisher exact test was used. For multiple group comparison, one-way ANOVA or Kruskal-Wallis followed by Tukey or Dunn’s post-hoc test was used, respectively, to correct for multiple testing. For non-independent data, nested one-way ANOVA was used. *P* < 0.05 was considered statistically significant.

## RESULTS

### CRISPR/Cas9 genome editing selectively removes pathogenic *Ryr2* allele

**Figure 1.**
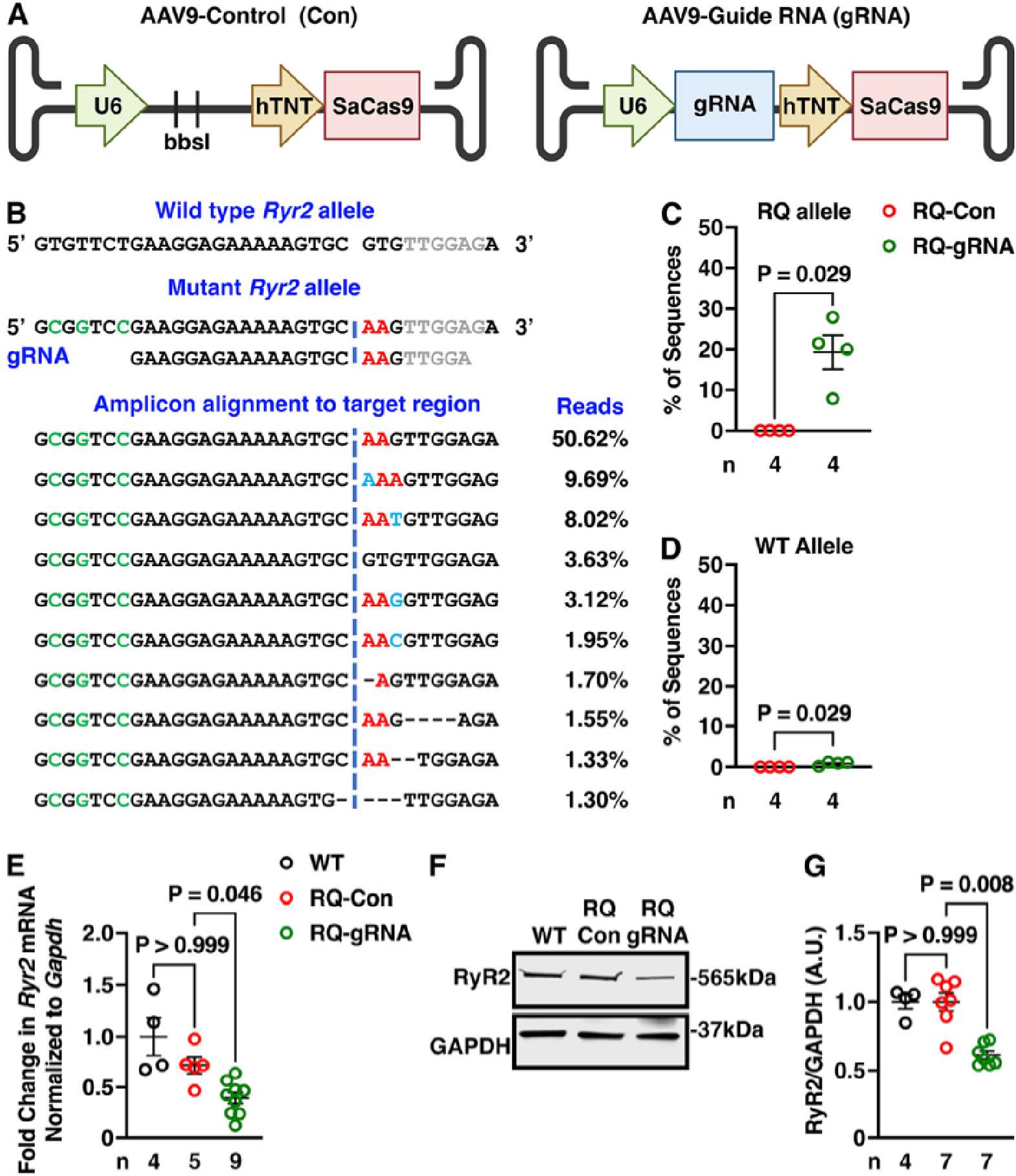
Allele-specific *Ryr2* gene editing leads to reduction of mutant protein in R176Q/+ mice. (A) Design of AAV vectors lacking insert at the BbsI cloning site (Con, control) and vector with guide RNA (gRNA) insert, packaged with U6 promoter for gRNA and human troponin T (hTNT) promoter for *Staphylococcus aureus* Cas9 (SaCas9) transcription. (B) Sequences of wild-type and mutant *Ryr2* allele, alignment with the gRNA, and most common variants detected in the mutant *Ryr2* allele of R176Q/+ mice using amplicon deep sequencing of cDNA at the pathogenic variant site (red). Insertions are shown in light blue. The silent restriction site in the mutant mouse allele (used for genotyping of the mice) is shown in green. (C) Percent of sequence reads with a mutation in the R176Q allele. (D) Percent of sequence reads with a mutation in the WT allele in R176Q/+ mice. (E) Quantification of *Ryr2* mRNA levels in WT, control, and gRNA-treated R176Q/+ mice. (F) Western blots and (G) quantification of RyR2 protein levels in WT, control, and gRNA-treated R176Q/+ mice. *P*-values based on the Mann-Whitney test (C and D), and the Kruskal-Wallis test followed by Dunn’s multiple comparison post-hoc test (E and G).

First, gRNA was designed for *Staphylococcus aureus* Cas9 (SaCas9) to edit the *Ryr2* g.GT528–527AA mutation site on the mutant allele. An AAV9 vector was used to drive the expression of the gRNA using the U6 promoter with a downstream SaCas9 driven by the human TNT promoter^[15]^. Another construct without gRNA and only containing Bbsl cloning sites was used as a control [Figure 1A]. Systemic gene delivery of the CRISPR/SaCas9 system was accomplished by injecting subcutaneously a single dose of 1.0 × 10^11^ vector genome per gram of AAV9 subcutaneously at P10 in cohorts of R176Q/+ heterozygous mice and WT littermate controls.

To determine the specificity and efficiency of the CRISPR Cas9 treatment regimen, amplicon-based next-generation sequencing (NGS) of the mutation site was performed using ventricular tissue of mice obtained 6 weeks after AAV9 injection [Figure 1B]. The majority of genome edits sequenced were small insertions and deletions (indels) 2–3 bp upstream of the mutation site [Figure 1B]. Approximately 85.4% ± 7.1% of these indels resulted in frame shifts. By using the upstream silent restriction site in the mutant allele as a barcode, the estimated genome editing frequency of the mutant (R176Q) allele was 19.3% ± 4.2% in R176Q/+ mice receiving gRNA-SaCas9 AAvV9 compared with 0.02% ± 0.02% for R176Q/+ mice receiving control AAV9 (*P* = 0.029; Figure 1C). In contrast, the off-target editing of the WT allele of R176Q/+ mice was very low (0.84% ± 0.23%), whereas there was no background editing (0.00% ± 0.00%) in R176Q/+ mice receiving control AAV9 [Figure 1D]. We then performed NGS amplicon sequencing on the target site using heart tissue obtained 6 weeks after the AAV9 injection of WT mice. Editing of the WT mice (that only possess the WT allele) treated with gRNA SaCas9 was negligible at 0.03% ± 0.02%, with 0.00% ± 0.01% editing in wild-type mice treated with AAV control.

One limitation of amplicon sequencing using short-range PCR is the inability to detect large insertions, deletions, or translocations, thereby underestimating editing efficiency. We determined that R176Q/+ heterozygous mice receiving control AAV9 had an average of 47.0% ± 0.3% mutant allele copies. We found that R176Q/+ mice that received AAV9 encoding gRNA-SaCas9 had a lower average of 32.4% ± 1.6% mutant allele copies. The 14.6% ± 1.6% (*P* < 0.001) decrease in mutant allele copies is likely due to editing-induced large gene modifications, including large insertions (> 50 bp) or large deletions longer than the size of the 227 bp amplicon.

Next, RT-qPCR revealed the impact of genome editing on the total *Ryr2* mRNA levels. The *Ryr2* mRNA levels in R176Q/+ mice receiving control AAV9 (0.99% ± 0.11%) were unaltered compared with WT controls (1.39% ± 0.25%, *P* > 0.999), while *Ryr2* mRNA levels were decreased to 0.55% ± 0.07% in R176Q/+ treated with gRNA-SaCas9 [Figure 1E]. In addition, western blotting was performed to quantify the effects of gRNA/Cas9 on RyR2 protein levels, using a RyR2 antibody with an epitope downstream of the mutation site. In agreement with the mRNA expression results, there was no significant decrease in RyR2 protein levels in R176Q/+ mice receiving control AAV9 (1.02% ± 0.06%) compared with WT controls (1.00% ± 0.05%, *P* ≥ 0.999). On the other hand, RyR2 protein levels were decreased to 0.61% ± 0.03% *vs*. WT controls (*P* = 0.008) in R176Q/+ receiving gRNA-SaCas9 AAV9 [Figure 1F and G].

**Figure 2.**
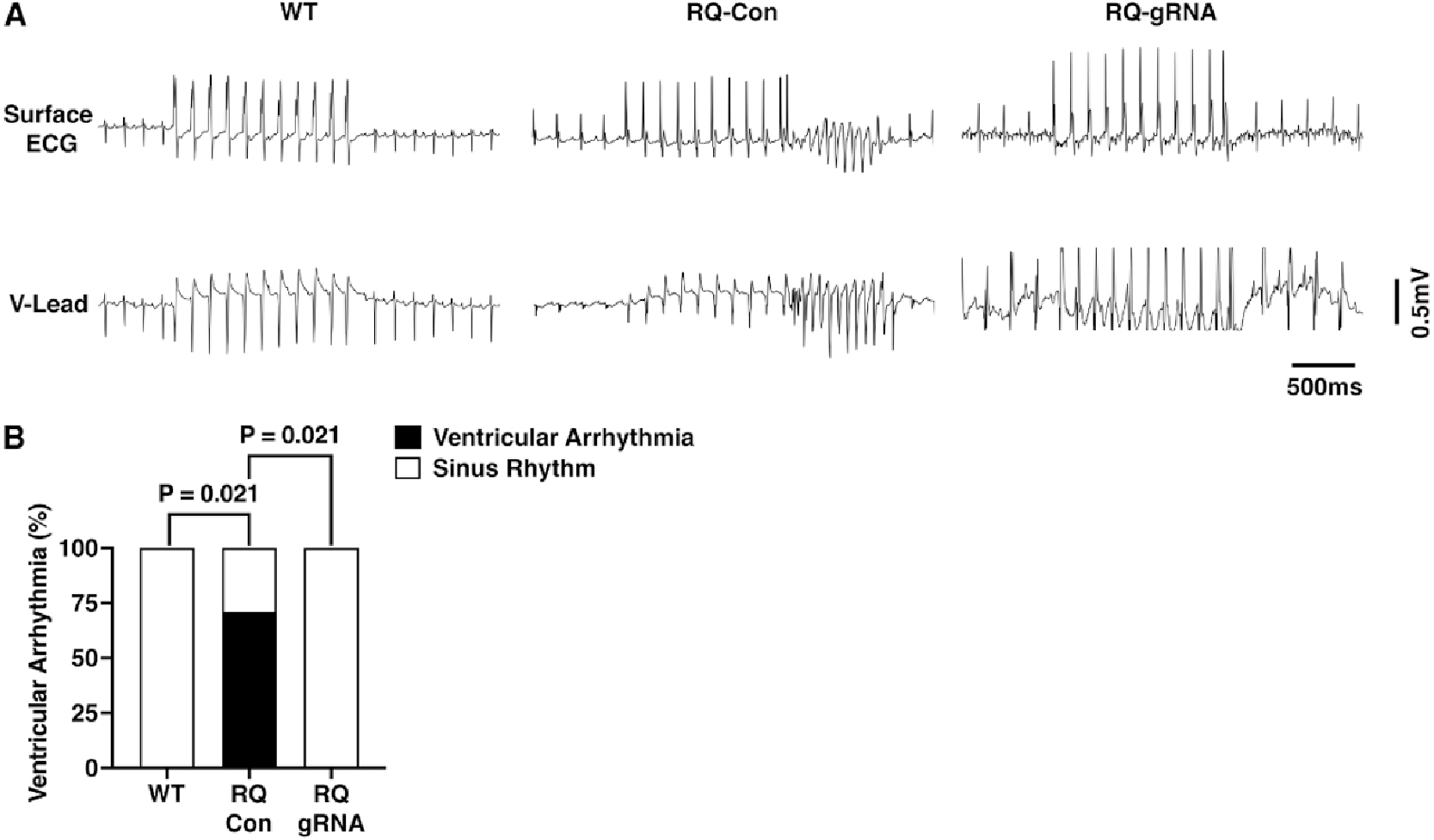
Prevention of stress-induced ventricular tachycardia in R176Q/+ mice at 6 weeks after AAV9. (A) Representative surface ECG (top) and intracardiac ventricular (V) lead (bottom) during programmed electrical stimulation (middle part of tracing). (B) Incidence of ventricular tachycardia induction in WT (*n* = 7 mice), RQ control (Con) (*n* = 8 mice), and RQ gRNA-SaCas9 (*n* = 7 mice) groups. The Fisher exact test was performed to compare for differences in arrhythmia induction (B).

Genome Editing Prevents Ventricular Arrhythmias in R176Q/+ Mice. To determine whether genome editing prevents the induction of ventricular tachyarrhythmias in R176Q/+ mice, programmed electrical stimulation was performed at 6 weeks after AAV9 administration. There were no significant differences in baseline rhythm by surface or intracardiac ECG patterns [Supplementary Figure 2] and intervals [Supplementary Table 1] comparing the three groups of mice (WT, R176Q/+ treated with control AAV9, R176Q/+ treated with gRNA-SaCas9 AAV9). Next, to mimic the adrenergic stimulation that evokes arrhythmias in CPVT patients, mice were intraperitoneally injected with caffeine (120 mg/kg) and isoproterenol (2 mg/kg). This led to an increase in heart rate in all groups of mice [Supplementary Table 1]. Although some parameters changed compared to baseline values, importantly, there were no significant differences between the three groups, except for a lower heart rate after ISO and associated longer PR and QT intervals in control R176Q/+ mice. Programmed electrical stimulation led to the induction of ventricular tachycardia in 6 of 8 R176Q/+ mice that received control AAV9, which was significantly more than 0 of 7 WT mice (*P* = 0.021; Figure 2A and B).

In contrast, 0 of 7 R176Q/+ mice treated with gRNA-SaCas9 developed pacing-induced VT, which was significantly less than control R176Q/+ mice (*P* = 0.021; Figure 2B). Of the 8 control R176Q/+ mice, 4 had bi-directional ventricular tachycardia and 2 had polymorphic ventricular tachycardia. These results demonstrate that mutation-specific disruption of the mutant R176Q/+ allele using AAV-mediated genome editing offers significant protection from VT.

### Genome edited normalizes calcium handling in ventricular cardiomyocytes of R176Q/+ mice

**Figure 3.**
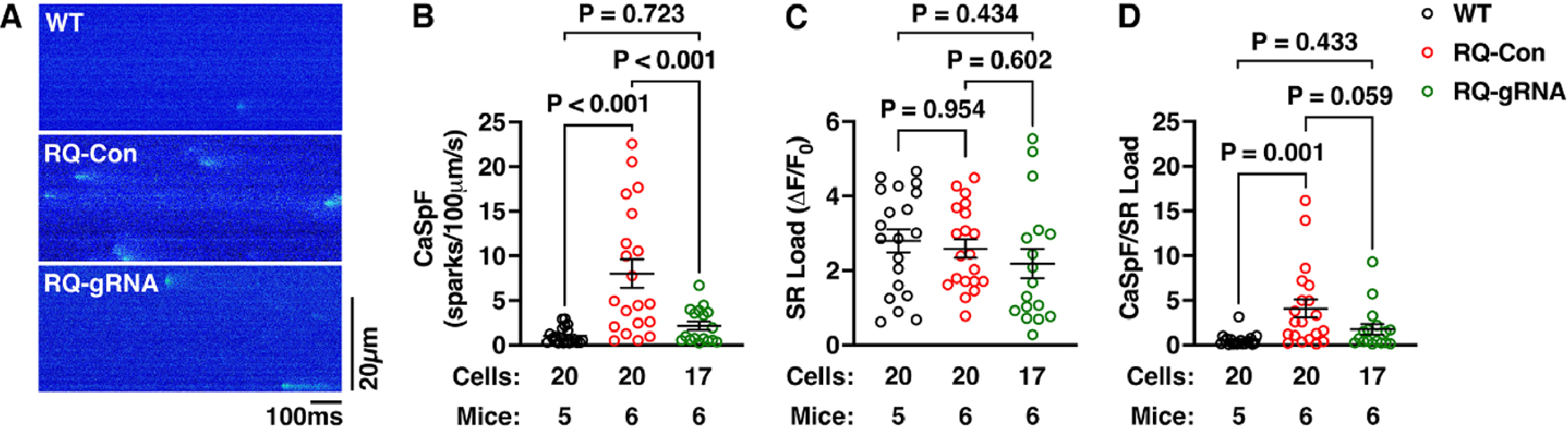
Normalization of diastolic SR Ca^2+^ release events due to genome editing. (A) Representative confocal line scan images showing sarcoplasmic reticulum (SR) Ca^2+^ sparks in ventricular myocytes isolated from R176Q/+ mice treated with control (Con) AAV9 or gRNA-SaCas9, or WT littermate mice. (B) Quantification of Ca^2+^ spark frequency (CaSpF), (C) SR Ca^2+^ leak measured using the caffeine dump protocol, and (D) ratio of Ca^2+^ spark frequency and SR Ca^2+^ load. Numbers of cells and mice are indicated. *P*-values are based on the nested one-way ANOVA test.

To assess the role of mutation-specific genome editing at the cellular level, ventricular myocytes were isolated from the hearts of R176Q/+ mice and WT littermates. Confocal line scan imaging revealed enhanced RyR2 activity evidenced by an increased Ca^2+^ spark frequency in ventricular myocytes isolated from control R176Q/+ mice (8.0 ± 1.6 sparks/100 mm/s) *vs.* WT mice (1.1 ± 0.2; *P* < 0.001; Figure 3A and B).

On the other hand, gRNA-SaCas9-treated R176Q/+ mice exhibited a significantly reduced Ca^2+^ spark frequency (2.2 ± 0.5; *P* = 0.001 *vs*. control R176Q/+; Figure 3A and B). A complete summary of all Ca^2+^ spark parameters is included in Supplementary Table 2. No differences were observed in the SR Ca^2+^ load as determined by the caffeine dump protocol comparing all three groups [Figure 3C]. Finally, the Ca^2+^ spark frequency normalized to cellular SR Ca^2+^ load was significantly elevated in ventricular myocytes from control R176Q/+ mice *vs.* those from WT mice, while there was a trend towards a reduction in the normalized Ca^2+^ spark frequency in gRNA-SaCas9 treated R176Q/+ mouse myocytes [Figure 3D]. These findings indicate that CRISPR/Cas9 genome editing targeting the R176Q/+ mutation and disrupting the mutant allele normalizes cellular Ca^2+^ handling, which is likely to be responsible for the observed antiarrhythmic effect.

### Long-term preserved cardiac contractile performance in genome-edited R176Q/+ mice

**Figure 4.**
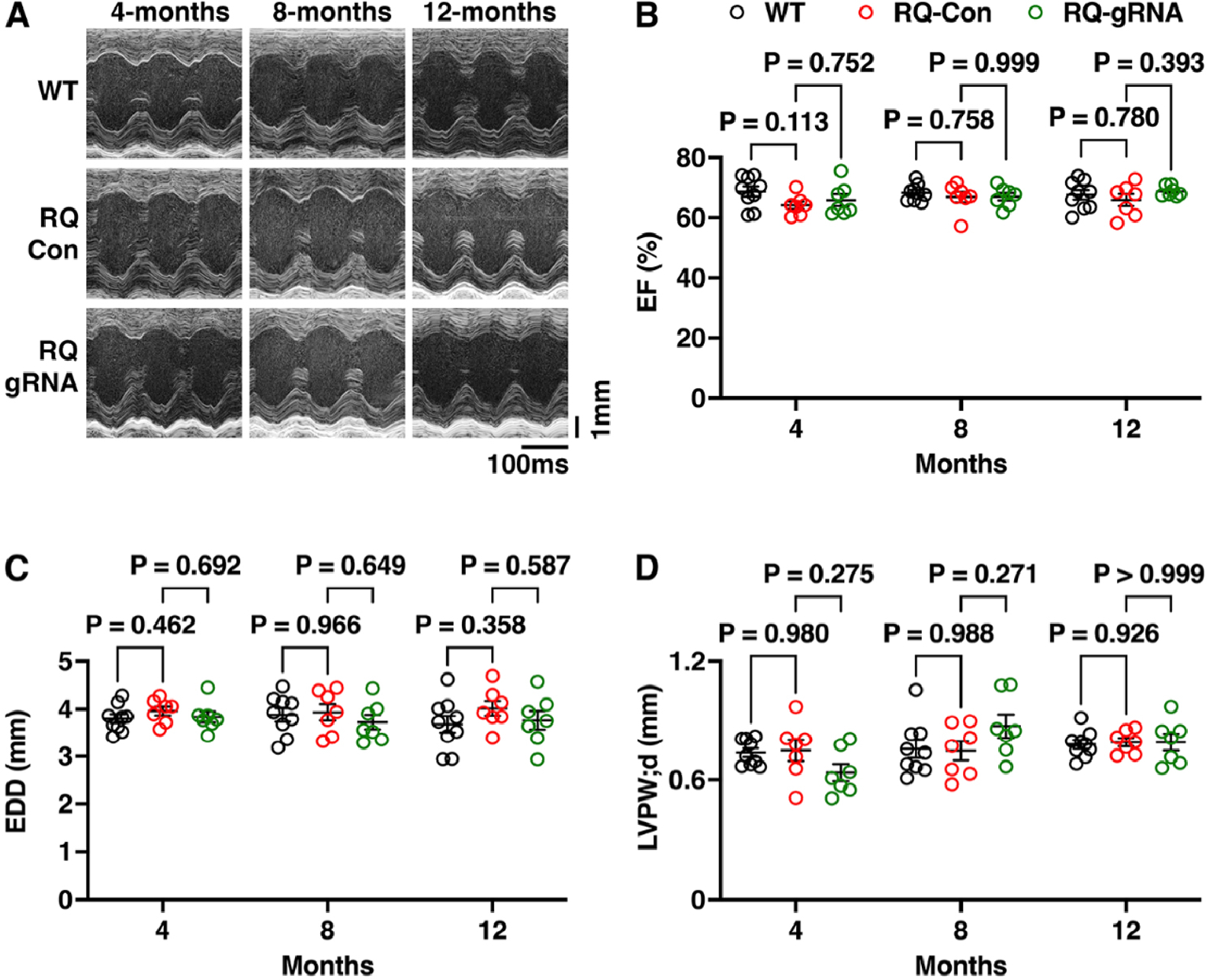
Unaltered cardiac function following long-term genome editing. (A) Representative short-axis M-mode echocardiography images of the left ventricle of R176Q/+ mice treated with control (Con) AAV9 or gRNA-SaCas9, or WT littermate mice, at 4, 8, and 12 months after AAV9 administration. Quantification of (B) ejection fraction, (C) end-diastolic diameter, and (D) left ventricular posterior wall thickness in diastole. *P*-values based on the one-way ANOVA test followed by Tukey’s post-hoc test.

To determine whether the reduction in total RyR2 levels because of genome editing would impact long-term cardiac dimensions or contractile function, we performed serial echocardiography studies on WT and R176Q/+ mice randomized to control or gRNA-SaCas9 AAV9 injection at p10 [Figure 4A]. There were no significant differences in ejection fraction [Figure 4B], end-diastolic diameter [Figure 4C], or left ventricular posterior wall thickness in diastole [Figure 4D] comparing the 4, 8, and 12-month post-AAV9 time points in any of the three groups (WT, R176Q/+ control, and R176Q/+ gRNA-Cas9 treated) [Figure 4B-D]. A complete summary of all echocardiography parameters in the three time points for the three groups of mice is provided in Supplementary Table 3. These findings reveal that mutation-specific targeting and disruption of the R176Q/+ allele is safe and does not impact long-term cardiac performance in genome-edited R176Q/+ mice.

### Long-term antiarrhythmic effects of genome editing

**Figure 5.**
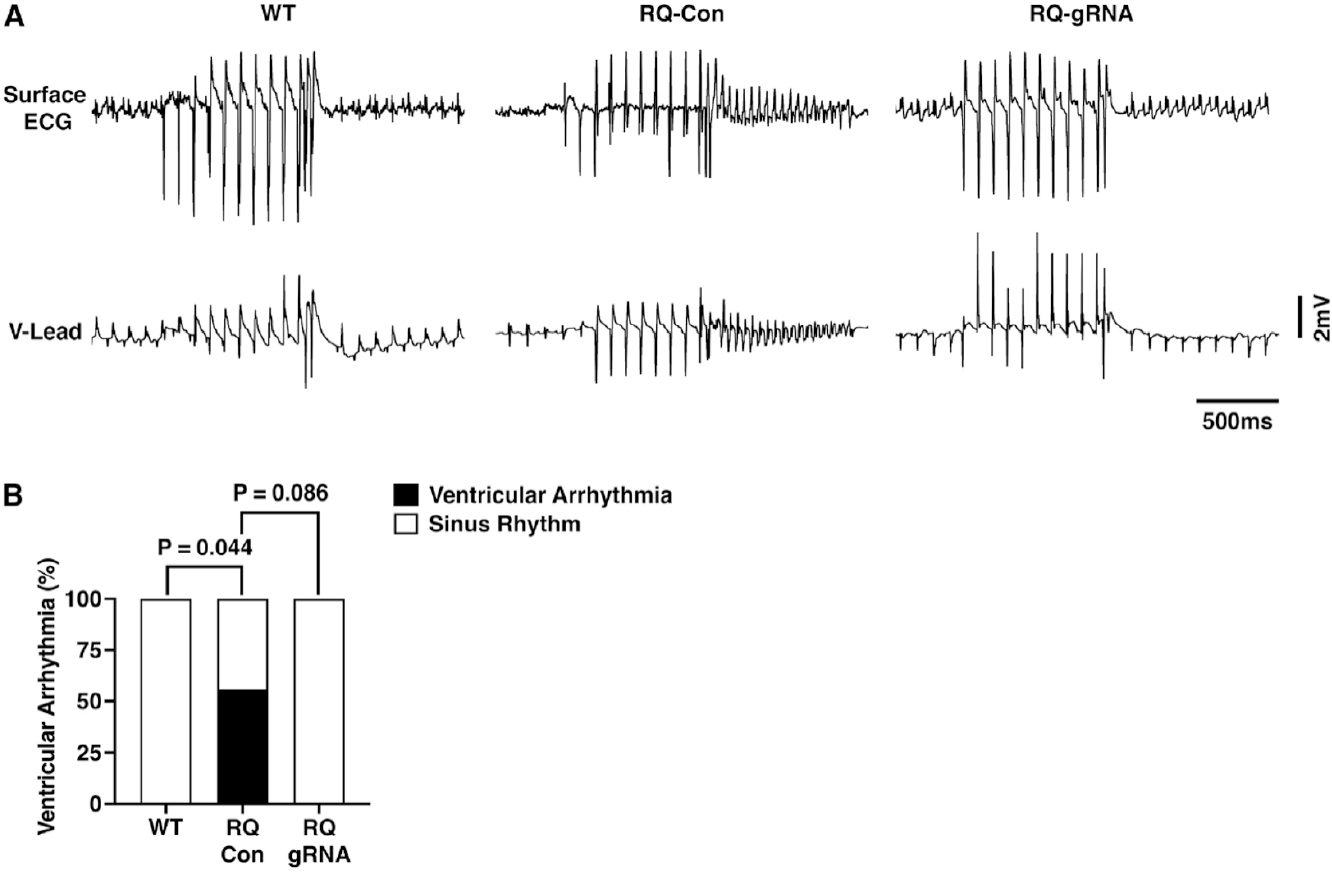
Prevention of stress-induced ventricular tachycardia in R176Q/+ mice at 12 months after AAV9. (A) Representative surface ECG (top) and intracardiac ventricular (V) lead (bottom) during programmed electrical stimulation (middle part of tracing). (B) Incidence of ventricular tachycardia induction in WT (*n* = 6 mice), RQ control (Con) (*n* = 7 mice), and RQ gRNA-SaCas9 (*n* = 5 mice) groups. The Fisher exact test was performed to compare for differences in arrhythmia induction (B).

To assess whether the antiarrhythmic consequences of CRISPR/Cas9 genome editing in R176Q/+ mice were long-lasting, programmed electrical stimulation was performed 12 months after AAV9 administration following the completion of the serial echocardiography studies. Like the observations made in mice 6-weeks after AAV9 injection, there were no significant differences in baseline ECG rhythm patterns [Supplementary Figure 3] and intervals [Supplementary Table 4] comparing the three groups of mice, with the exception of a relatively minor but significant difference in heart rate in control R176Q/+ mice at baseline and in treated R176Q/+ mice after isoproterenol. Programmed electrical stimulation before the intraperitoneal injection of isoproterenol (2 mg/kg, IP) and caffeine (120 mg/kg, IP) led to the induction of ventricular tachycardia in 4 of 7 R176Q/+ mice that received control AAV9, which was significantly more than 0 of 6 WT mice (*P* = 0.044; Figure 5A and B). In contrast, 0 of the 5 R176Q/+ mice subjected to gRNA-SaCas9 treatment developed VT following pacing protocols (*P* = 0.086 *vs*. R176Q/− controls; Figure 5B). Of the 7 R176Q/+ mice, 3 had bidirectional ventricular tachycardia and 1 had polymorphic ventricular tachycardia. Thus, the protective effects of genome editing against pacing-induced ventricular tachycardia are long-lasting in R176Q/+ mutant mice.

### Absence of adverse cardiac remodeling after long-term genome editing

**Figure 6.**
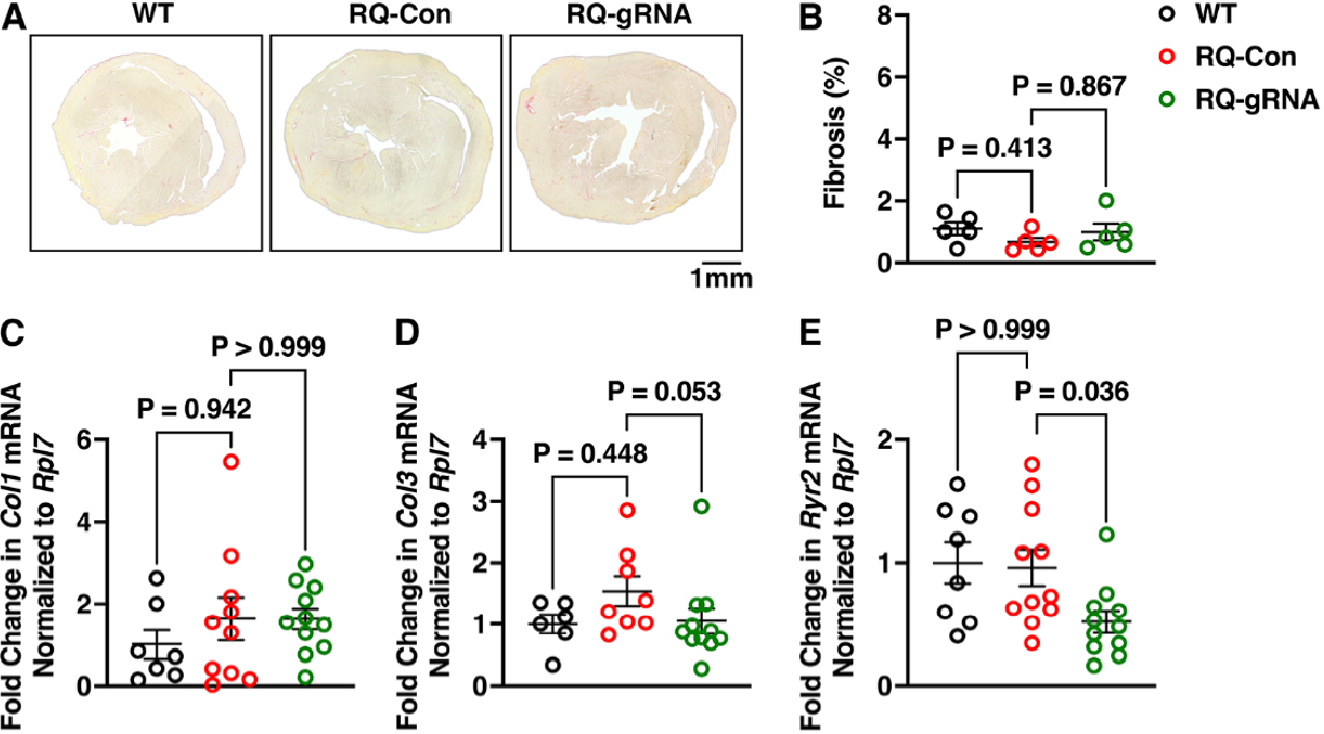
Absence of tissue remodeling after long-term genome editing in R176Q/+ mice. (A) Picrosirius staining of transverse sections of heart from R176Q/+ mice treated with control (Con) AAV9 or gRNA-SaCas9, or WT littermate mice, at 12 months after AAV9 administration. (B) Quantification showing unaltered levels of cardiac fibrosis. (C) RT-PCR showing reduced levels of *Ryr2* mRNA in hearts of R176Q/+ mice treated with gRNA-SaCas9. (D) Representation of western blots and (E) quantification thereof showing reduced RyR2 protein levels in hearts of R176Q/+ mice treated with gRNA-SaCas9. *P*-values based on the Kruskal-Wallis test followed by Dunn’s multiple comparison post-hoc test.

A set of experiments was performed to assess whether AAV9-mediated genome editing was possibly associated with adverse heart and/or liver tissue remodeling. Histological transverse sections of the hearts were stained using hematoxylin and eosin (H&E) staining [Supplementary Figure 4A]. Cardiac structure and dimensions were normal at the 1× magnification. Moreover, there were no signs of tissue disarrangement or cellular infiltrates at 40× magnification [Supplementary Figure 4B]. Picrosirius staining revealed similar, low levels of fibrosis in the hearts of WT, R176Q/+ control, and R176Q/+ gRNA-Cas9 treated mice [Figure 6A and B]. Because AAV9 is known to accumulate in the liver following systemic delivery^[16]^, liver sections were also stained using H&E. There were no signs of tissue disarray or immune cell infiltration. RT-PCR was done to quantify the expression levels of collagen−1 and −3 in the hearts of the 3 groups of mice. Normalized *Col1a1* mRNA levels in R176Q/+ mice treated with gRNA-Cas9 were similar (1.63 ± 0.24) to control treated R176Q/+ mice (1.63 ± 0.48; *P* > 0.999) and WT mice (1.00 ± 0.31; *P* > 0.999) [Figure 6C]. Similarly, unaltered *Col3a1* mRNA expression levels were observed [Figure 6D]. Finally, we set out to determine whether the genome editing effects on RyR2 expression levels were sustained at 12 months after AAV9 injection. RT-PCR revealed no significant decrease in *Ryr2* mRNA levels in R176Q/+ mice receiving control AAV9 (0.96% ± 0.14%) *vs*. WT controls (1.00% ± 0.16%, *P* > 0.999), while there was a significant decrease in *Ryr2* mRNA levels (0.52% ± 0.09%; *P* < 0.036) in R176Q/+ treated with gRNA-SaCas9 [Figure 6E]. Likewise, SaCas9 mRNA expression remained elevated 12 months after gRNA-SaCas9 treatment [Supplementary Figure 5]. Thus, genome editing effects on RyR2 were persistent up to at least 12 months after AAV9 injection, in the absence of any overt cardiac or liver tissue remodeling effects.

## DISCUSSION

Genome editing is a potential therapeutic option for many cardiovascular disorders including monogenetic arrhythmia syndromes. There is a growing acceptance of somatic gene editing therapies and numerous companies are pursuing preclinical and even clinical programs in the cardiovascular space. Understanding the efficacy and off targets of each genome editing approach is crucial for future iterations. We have previously shown that genome editing of the autosomal-dominant arrhythmia disorder CPVT is an attractive therapeutic approach because of the high efficiency of CRISPR-Cas9 genome editing in disrupting mutant allele expression. While our prior studies utilized a gRNA targeting an artificial silent restriction site introduced when generating the mutant knock-in allele^[12]^, in the current study, we were able to target the actual mutation site *Ryr2* GT527–528AA using a different gRNA. Our data reveal efficient editing at the target site with low levels of off-target genome editing. We also, for the first time, established that the ~40% decrease in total RyR2 protein levels did not lead to adverse remodeling of the heart over a 12-month period after genome editing. However, the beneficial effects of genome editing persisted as R176Q/+ mutant mice remained protected from isoproterenol and pacing-induced ventricular tachyarrhythmias. Thus, our preclinical studies provide evidence for the long-term efficacy and safety of cardiac genome editing for the treatment of CPVT.

While several nucleases can be used to insert double-stranded DNA breaks (DSBs) at defined locations in the genome for editing purposes, only the CRISPR/Cas9 system has been exploited for therapeutic purposes, i.e., somatic genome editing for cardiac disorders^[17]^. Here, we used a single AAV9 vector to deliver a gRNA to target Cas9 to the target genomic sequence containing the disease-causing mutation. Consistent with prior studies^[12,18]^, we showed that the DSBs are mainly repaired using non-homologous end-joining (NHEJ). The lack of reliability in NHEJ, however, often results in insertions and deletions (indels) in the mutant *Ryr2* allele that can lead to frame shifts, which in turn may result in nonsense-mediated degradation^[19]^. Indeed, we observed reduced *Ryr2* mRNA and RyR2 protein levels in mutant R176Q/+ mice following genome editing. Deep sequencing revealed that the indels occurred in the R176Q/+ mutant allele with very low levels of editing in the WT allele of R176Q/+ mice or WT littermates that received gRNA-SaCas9 treatment. The high specificity is most likely a result of the mutation sequence being near the 3′-end of the guide RNA.

Nevertheless, a potential side-effect of genome editing is an excessive reduction of the target protein levels. In the case of RyR2, it has been somewhat controversial whether a 50% reduction in RyR2 has an impact on cardiac function. Mice that were heterozygous for RyR2, in which total RyR2 levels were reduced to about 40% of control levels, were viable and did not exhibit an increased mortality rate^[20]^. Additional studies revealed no differences in the morphology of cardiomyocytes and normal contractility in RyR2 heterozygous mice^[21]^. In addition, heterozygous RyR2 knockout in rabbits also did not cause an overt phenotype at the whole animal and whole heart level, although subcellular rearrangements of RyR2 clusters were observed^[22]^. On the other hand, > 50% reductions in RyR2 are more likely associated with heart failure and increased mortality^[20,23]^. Moreover, loss-of-function *RYR2* variants can cause a distinct arrhythmia syndrome known as ‘calcium release deficiency syndrome’ (CRDS)^[24]^. Thus, as long as the genome editing-associated reduction in RyR2 levels is limited to the mutant allele and the total RyR2 levels do not drop below 50%, this therapeutic strategy is expected to be well tolerated without an impact on contractility or adverse remodeling.

The functional effect of reducing the level of mutant RyR2 was the stabilization of SR Ca^2+^ handling in ventricular myocytes isolated from R176Q/+ heterozygous mice. Our studies were consistent with prior observations showing similar effects on preventing the increase in SR Ca^2+^ spark frequency seen in control treated R176Q/+ mice^[12]^. However, prior studies did not establish the long-term safety and efficacy of this genome-editing approach. Characterization of the R176Q/+ mice post-genome editing revealed the absence of cardiac ventricular arrhythmias and stable cardiac function for up to 12 months post-injection. These studies suggest that the reduction in excessive SR Ca^2+^ sparks in genome-edited R176Q/+ mice did not impair cardiac contractility, as evidenced by serial echocardiography studies. Moreover, ECG parameters remained unaltered in genome-edited R176Q/+ mice up to 12 months after AAV9 administration. Finally, there was no evidence that the long-term expression of gRNA and SaCas9 induced any detrimental remodeling in cardiac or liver tissue. Taken together, our studies provide evidence for the long-term safety of AAV9-mediated CRISPR/Cas9-mediated genome editing for the treatment of CPVT in a mouse model.

One limitation of this study is that a rodent model of CPVT was used. Adult mouse cardiomyocytes have short action potentials and are more tolerant to calcium loading compared to human myocytes^[25]^. On the other hand, the function of RyR2 is relatively conserved among species, and generally, findings obtained in ventricular myocytes isolated from RyR2 mutant mice have been replicated in human CPVT patient-derived induced pluripotent stem cell cardiomyocytes^[10,26]^. Another limitation is that our studies most likely underestimated the level of genome editing, as our next generation sequencing only covered the 227 bp amplicon. This means that any deletion larger than 227 bp, any translocation, large AAV genome integrations, or chromosomal rearrangement would not get amplified in the PCR reaction preceding the NGS. Likewise, while we chose our guide to have 0 off targets with < 3 mismatches *in silico*, our *in vivo* analysis of off-target effects was limited to the amplicon region of the wild-type allele. Finally, the current genome editing approach requires a unique gRNA for each RyR2 variant that needs to be corrected. In addition, future therapies that can correct RyR2 mutations without reducing total RyR2 levels might be preferable.

An important consideration for future CRISPR/Cas9-based therapies is whether the effectiveness of genome editing will translate to older adults. Most preclinical studies in animals have applied treatments at younger ages and then followed these animals to older ages. For example, a recent study on PCSK9 gene editing for hypercholesteremia has shown the durability and effectiveness of liver genome editing starting at 9 weeks of age until 476 days after treatment in non-human primates^[27]^. The clinical trial for the same PCSK9 therapy is currently recruiting for ages ranging up to 75 [NCT05398029] and will reveal the relative effectiveness depending on the starting age of the therapy or whether there will be immune reactions to SaCas9 specific to older adults. For the translation of our approach disrupting mutant RyR2 expression, one concern with older adults is that RyR2 levels may decrease with comorbidities such as diabetes^[28]^ and heart failure^[29]^, in which case a RyR2 replacement therapy might be needed.

In summary, gene therapy is a promising treatment modality for monogenetic arrhythmia disorders when the genetic cause of the disease is clearly defined. In this manuscript, we have shown that by specifically targeting the mutation site R176Q on *Ryr2* in a mouse model of CPVT, we could prevent arrhythmogenesis without significant long-term effects on baseline cardiac function. In particular, patients with CPVT are dependent on consistently taking daily medications. Additionally, they cannot rely on an ICD to protect them. Hence, a single missed dose could potentially be life-threatening. Thus, a treatment strategy that provides long-term protective effects is highly desirable for this disease. These studies provide new evidence that long-term AAV9-mediated CRISPR/Cas9 genome editing can be safe and efficacious, at least in a preclinical animal model of an inherited arrhythmia syndrome.

## Supplementary Material

Suplementary Material

## Data Availability

Original data sets are available from the corresponding author upon request.
