# Supplementary material for: Long-term efficacy and safety of cardiac genome editing for catecholaminergic polymorphic ventricular tachycardia
Source: J Cardiovasc Aging. Author manuscript; Available in PMC 2025 Jan 4. (PMC10919902; doi:10.20517/jca.2023.42)
Supplement: Suplementary Material [file NIHMS1962962-supplement-Suplementary_Material.docx]

**Supplemental Materials**

**Long-term Efficacy and Safety of Cardiac Genome Editing for Catecholaminergic Polymorphic Ventricular Tachycardia**

Oliver M. Moore^1,2,3^, Yuriana Aguilar-Sanchez^1,2^, Satadru K. Lahiri^1,2^, Mohit M. Hulsurkar^1,2^, J. Alberto Navarro-Garcia^1,2^, Tarah A. Word^1,2^, Joshua A. Keefe^1,2^, Dean Barazi^1,2^, Elda M. Munivez^1,2^, Charles T. Moore^1,2^,Vaidya Parthasarathy^1,2^, Jayso’n Davidson^1,2^, William R. Lagor^1,2^, So Hyun Park^4^, Gang Bao^4^, Christina Y. Miyake^5^, Xander H.T. Wehrens^1,2,3,5,6,7^

^1^Cardiovascular Research Institute, ^2^Department of Integrative Physiology, ^3^Department of Neuroscience, Baylor College of Medicine, Houston, TX 77030, USA, ^4^Department of Bioengineering, Rice University, Houston, TX 77030, USA, ^5^Department of Pediatrics (Cardiology), ^6^Department of Medicine (Cardiology), ^7^Center for Space Medicine, Baylor College of Medicine, Houston, TX 77030, USA

**Supplemental Methods**

**Adeno-Associated Virus (AAV) Plasmids.** The AAV-CRISPR control vector, pAAV-U6-SA-BbsI-TNT-SACas9-HA-OLLAS-spA was generated through subcloning of the human TNT promoter into plasmid 109320 (Addgene, Watertown, MA). The AAV-CRISPR vector contains a site for cloning custom gRNA. CRISPR SaCas9 guide RNA (gRNA) was designed to target the *Ryr2* g.GT528-527AA mutation site. The guide was chosen based on its proximity to a SaCas9 recognition sequence, NNGRR, and containment of the mutation site within the 3’-end of the gRNA to minimize off target editing of the wild type allele. Oligos for the gRNA were annealed and cloned into the BbsI restriction digest site using the following oligo sequences: 5’-CACC gCCGAAGGAGAAAAAGTGCAAG-3’, and 5’-AAAC CTTGCACTTTTTCTCCTTCGGc-3’. The final guide RNA vector pAAV-U6-SA-gRNA-TNT-SACas9-HA-OLLAS-spA was Sanger sequenced at the cloning sites, and the inverted terminal repeats (ITR) was verified by restriction digest. Sequences of plasmids used to generate the AAVs are listed in Supplemental Figure 1.

**Adeno-Associated Virus type 9 (AAV9) Production and Purification.** AAV9 were packaged in the HEK293T cells (ATCC) by the triple transfection method of Xiao *et al.*^[1]^. The Adenoviral helper plasmid pAdDeltaF6 (PL-F-PVADF6) and the AAV9 packaging vector pAAV2/9 (PL-T-P0008-R2) were obtained from the University of Pennsylvania Vector Core. Large-scale plasmid preps of these packaging vectors were generated by Puresyn, Inc (Malvern, PA). The control and guide RNA vectors described above were used for the AAV production. Briefly, 12 hours before transfection, cells were seeded in 150 mm plates (30-50% confluent) fed with DMEM (#BE15-604D, Lonza, Morristown, NJ) containing 10% fetal bovine serum (#A5670701, Gibco, Grand Island, NY) with L-Glutamine and Penicillin/Streptomycin (#10378016, Gibco, Grand Island, NY).

After 48-72 hours, cells were processed by TrypLE (#12604013, Gibco, Grand Island, NY), collected in PBS (#10010023, Corning, Corning, NY) and resuspended in resuspension buffer (50 mM Tris–HCl pH 8.0, 150 mM NaCl, 2 mM MgCl_2_). The cells were subjected to 3 freeze-thaw cycles in -80°C for 10 minutes, 37°C for 20 minutes. Cells were added with 3,000 U Benzonase (#9025-65-4, MilliporeSigma, Burlington, MA) and incubated at 37°C for 1 hour to digest cellular genomic DNA. The suspension was treated with 1/39^th^ volume of 1M CaCl_2_ solution and 2/3 volume of 20% PEG 8000/1.25N NaCl to remove the cell debris and precipitate AAV. AAV was then resuspended in HBS and purified by CsCl_2_ gradient. After a round of CsCl_2_ gradient in 45,000 rpm and 60,000 rpm, fractions were collected according to the desired refractive index, respectively, 1.3680-1.3750 for the first gradient and 1.3680-1.3750 for the second gradient.

AAV were dialyzed against PBS in 10,000 MWCO Slide-A-Lyzer Cassettes (#A52971, ThermoFisher, Waltham, MA), and concentrated using Amicon 100 kDa MWCO centrifugal filtration device (# UFC910008, MilliporeSigma, Burlington, MA) prior to storage at -80°C. AAVs were tittered following DNAse digestion and using ABM’s qPCR AAV Titer Kit (G931, ABM, Richmond, BC). AAV transgene plasmids were serially diluted to generate standard curves, and the genomic copies numbers per mL were calculated for each virus ^[2]^.

**Next-Generation Deep Sequencing.** Total genomic DNA was isolated from ventricular tissues by TRIzol (#15596, Life Technologies, Carlsbad, CA) and reverse transcribed by iScript (#1708841, Bio-Rad, Hercules, CA). *Ryr2* genomic DNA was amplified with primers targeting upstream and downstream of the mutation site by Phusion High-Fidelity DNA Polymerase (#M0530S, New England Biolabs, Ipswich, MA). Primers used: Fw: 5’-CGTCATCTGGAGAGGGAATG-3’, Rev: 5’-CTCTCAGCAGAGCATCAAGC-3’. Adapters were added through PCR. All amplicons were purified using the Wizard PCR Clean-up system (#A9282, Promega, Madison, WI). Samples were sent to Azenta (Burlington, MA) for next generation amplicon sequencing.

Paired-end Illumina reads were merged using FLASH (Fast Length Adjustment of Short reads) ^[3]^. WT and mutant alleles were filtered based on WT and mutant allele-specific SNPs using SeqKit ^[4]^. WT or mutant reads were analyzed separately for editing frequency using CRISPResso2 ^[5]^. Percentages of WT or mutant reads, and Indel frequency on WT or mutant reads were reported, respectively.

**RT-PCR**. Total RNA was isolated from ventricular tissues by TRIzol (#15596, Life technologies, Carlsbad, CA) and was reverse transcribed by iScript (#1708841, Bio-Rad; Hercules, CA). The iTaq Universal SYBR Green Supermix (Thermo Fisher Scientific, Waltham, MA) was used for the quantitative polymerase chain reaction (PCR) analysis of diluted cDNA in 1:10. The ΔΔCT method was used to calculate relative quantities normalized to L7. Primers used: RyR2 Fw: 5’-TCAAACCACGAACACATTGAGG-3’, RyR2 Rev: 5’-AGGCGGTAAAACATGATGTCAG-3’, Jph2 Fw: 5’-ACTCTGGCTCCTGGAACTTTG-3’, Jph2 Rev: 5’-GCGCCCCTTGGTCTCTATG-3’, RPL7 Fw: 5’-AAGATCAAGCGCCTGAGAAAG-3’, RPL7 Rev: 5’-TGCAGGTACATAGAAGTTGCCA-3’, SaCas9 Fw: 5’-GTACTACGAGGAAACCGGGAAC-3’, SaCas9 Rev: 5’-GTTGTTGTAGAAGGAGGCGATAAAC-3’, U6 Fw: 5’- CCTTCATATTTGCATATACGATACAAGG CTGTTAG-3’.

**Western Blotting**. Ventricular heart samples were crushed in liquid nitrogen and resuspended in RIPA buffer containing 1% CHAPS, Phos-STOP (#4906837001, Sigma-Aldrich; St. Louis, MO) and complete mini protease inhibitor cocktail (#4693124001, Sigma-Aldrich; St. Louis, MO), 20mM sodium fluoride (NaF), 1mM Na_3_VO_4_. Lysates were further homogenized with steel beads using a homogenizer (Tissue Lyser LT; Qiagen, Germantown, MD) at 50-Hz for 8 minutes. These samples were sonicated 3 times for 2 seconds each and centrifuged at 14,000 rpm for 20 mins at 4°C. Supernatants were collected as lysates, and protein concentration was measured using a NanoDrop spectrophotometer (Thermo Fisher Scientific, Waltham, MA). Human heart, mouse heart, or cell lysates (75 mg) were denatured in 2x Laemmli sample buffer (#1610737, Biorad; Hercules, CA) with 5% 2-mercaptoethanol (#M3148, Sigma-Aldrich; St. Louis, MO) for 10 mins at 70°C. SDS gel electrophoresis was done on 10-12% acrylamide gel at 100 Volts. Proteins were further transferred onto 0.45-micron polyvinylidene fluoride (PVDF) membranes for 1.5-h at 100 Volts. Membranes were blocked for 1-h at room temperature and incubated overnight at 4°C with primary antibodies diluted in blocking buffer. Primary antibodies used were RYR2 (1:2000, Custom, Yenzyme, Rabbit), and GAPDH (1:10,000; mouse monoclonal; #MAB374, EMD Milipore, Burlington, MA). Membranes were washed 3 times with TBST (0.1% tween-20) for 10 minutes each and incubated with secondary antibody (1:10,000) for 1-h at room temperature. Secondary antibodies used were anti-mouse-Alexa-Fluor-680 (1:10,000, goat polyclonal; #A-21057, Thermo Fisher Scientific, Waltham, MA) and anti-rabbit-IR800 (1:10,000, goat polyclonal; #A-32735, Thermo Fisher Scientific, Waltham, MA). After washing 3x10 minutes in TBST, membranes were developed using LICOR Odyssey infrared imager (LICOR, Lincoln, NE). Bands were quantified using the ImageJ software and normalized to GAPDH levels.

**Programmed Electrical Stimulation (PES) Studies.** Electrophysiology studies were performed in R176Q/+ mice and wildtype littermates at 6-weeks or 12-months post AAV9 injection, as described ^[2]^. First, anesthesia was induced by placing the mouse inside an induction chamber filled with 2% isoflurane (#029405, Covetrus, Houston, TX) in 100% O_2_. Following induction, the mouse was transferred to a heated platform (Indus Instruments, Houston, TX) where the body temperature was maintained at 36.5-37.0°C and anesthesia (1.5% isoflurane in 100% O2 was maintained via a nose cone). The surface ECG was monitored using electrodes embedded in the heated board, connected to the external stimulator in recording mode (STG3008, MultiChannel Systems, Reutlingen, Germany) and acquired using the IOX2.4 acquisition software (Emka Technologies, Sterling, VA). A 0.5-inch incision was created to the right of the midline at the level of the clavicle. Then, subcutaneous tissue, salivary glands, and lymphatic tissues were separated with the help of blunt forceps to expose the right jugular vein. A tunnel was made under the jugular to cross a 6-0 suture to tie the proximal end of the vein and place another suture at the distal end of the visualized edge. While pulling gently on the suture to keep it straight, a small incision was made with the help of micro-scissors in the jugular and a 1.1 F octapolar catheter (EPR-800, Millar, Houston, TX) was inserted through it. The catheter was gently pulled, and the proper position of the catheter was verified by the waveforms of the 4 intracardiac electrocardiograms, as described ^[6]^.

Baseline ECG parameters (RR, HR, PR, QRS, and QTc) were measured in R176Q/+ mice and WT littermates that were treated with either control virus (AAV9-SaCas9/Bbsl) or editing virus (AAV9-SaCas9/gRNA). To mimic adrenergic stimulation, mice were injected with isoproterenol (2 mg/kg, i.p.) and caffeine (120 mg/kg, i.p.). To determine the inducibility of ventricular arrhythmias (VA) after adrenergic stimulation, programmed intracardiac stimulation was performed 10 minutes after the injections. All mice were subjected to the S1-S2 extra stimuli pacing at cycle length of 70 ms. In some mice, protocols were repeated at 90 ms cycle length. VA was defined as greater than 10 consecutive ventricular beats at a rate faster than 600 bpm. The incidence of inducible VA was calculated by the number of mice with reproducible VA divided by the total number of mice studied in each group.

**Calcium Imaging Studies.** Mouse hearts were excised, cannulated, and perfused retrogradely using a heated Langendorff system via the aorta, as described ^[7]^. The perfusion buffer consisted of Ca^2+^-free Tyrode solution containing (mM): 140 NaCl, 5.4 KCl, 1 MgCl_2_, 5 HEPES, and 10 Glucose, pH 7.4, supplemented with 22µg/mL Liberase TH (#5401119001, Sigma-Aldrich, St. Louis, MO). After digesting, hearts were perfused with 5mL Kraft-Brühe (KB) solution containing (mM): 90 KCl, 30 K_2_HPO_4_, 5 MgSO_4_, 5 pyruvic acid, 5 B-hydroxybutyric acid, 5 creatine, 20 taurine, 0.5 EGTA, 5 HEPES, 10 glucose, pH 7.2. Left ventricular tissue was teased apart and strained through a 250µm nylon mesh to isolate single cells. Ventricular cardiomyocytes were re-adapted to 1.8mM Ca^2+^ as described^[8]^, and loaded for 30 min with 2µM Fluo-4-AM (F14201, Invitrogen, Calsbad, CA) for Ca^2+^ imaging. Cells were plated onto laminin-coated (#354232, Corning, Corning, NY) coverslips and imaged using an LSM880 confocal microscope (Carl Zeiss, Thornwood, NY, USA) in line scan mode with 1024 pixels per line at 813Hz acquisition using 40X objective. Cardiomyocytes were paced at 1-Hz for 15s, unstimulated for the 60s, and then perfused with 10mM caffeine to assess SR load. Ca^2+^ transient amplitude and kinetics were analyzed using pCLAMP10 (Molecular Devices, San Jose, CA, USA). The Ca^2+^ spark frequency (CaSpF) was analyzed using the SparkMaster ImageJ plugin ^[9]^.

**Echocardiography**. Chest hair was removed using Nair cream before imaging. Mice were then anesthetized with an isoflurane concentration ranging from 1.5% to 2.0% mixed with 100% oxygen. To keep their body temperatures within a physiological range (36.5°C to 37.5°C), mice were placed on a temperature-regulated platform, and body temperature was monitored using a rectal probe ^[10]^. Cardiac function was assessed through ultrasound echocardiography, using both the Vevo 2100 and Vevo F2 systems (Fujifilm VisualSonics, Toronto, ON, Canada) with a probe operating at 30 MHz and 57 MHz respectively. Both B-mode and M-mode images were recorded in short and long-axis views of the hearts. Short-axis M-mode echocardiograms were subsequently analyzed with the VevoLab software to measure systolic function and cardiac chamber dimensions. Long-axis B-mode images were analyzed to assess left atrial size.

**Supplemental Table 1. ECG parameters at baseline and after isoproterenol at 6 weeks post-AAV9.**

| **Baseline** | **WT** | **RQ-Con** | **P (WT vs. RQ-Con)** | **RQ-Edited** | **P (RQ-Con vs. RQ-gRNA)** |
| --- | --- | --- | --- | --- | --- |
| N (mice) | 7 | 7 |  | 7 |  |
| HR (bpm) | 524.8 ± 16.6 | 526.3 ± 11.9 | 0.999 | 517.9 ± 11.0 | 0.999 |
| P wave (ms) | 16.1 ± 0.82 | 17.1± 1.11 | 0.999 | 18.1 ± 0.67 | 0.638 |
| PR interval (ms) | 38.4 ± 0.78 | 36.9 ± 1.19 | 0.999 | 36.6 ± 2.55 | 0.999 |
| QRS interval (ms) | 13.4 ± 0.34 | 12.5 ± 0.52 | 0.601 | 11.2 ± 0.31 | 0.192 |
| QT interval (ms) | 47.8 ± 1.37 | 52.9 ± 2.49 | 0.304 | 57.6 ± 2.54 | 0.633 |
| QTc interval (ms) | 44.6 ± 1.32 | 49.5 ± 2.16 | 0.363 | 53.4 ± 2.16 | 0.589 |

| **ISO + Caffeine** | **WT** | **RQ-Con** | **P (WT vs. RQ-Con)** | **RQ-Edited** | **P (RQ-Con vs. RQ-gRNA)** |
| --- | --- | --- | --- | --- | --- |
| N (mice) | 7 | 7 |  | 7 |  |
| HR (bpm) | 699.0 ± 9.3 | 588.8 ± 10.6 | **>0.001** | 663.0 ± 16.8 | 0.036 |
| P wave (ms) | 15.1 ± 0.98 | 15.1 ± 1.10 | 0.999 | 15.6 ± 1.33 | 0.999 |
| PR interval (ms) | 32.4 ± 0.54 | 40.1 ± 1.35 | **0.001** | 34.2 ± 0.66 | **0.042** |
| QRS interval (ms) | 11.8 ± 0.52 | 11.3 ± 0.32 | 0.823 | 12.1 ± 0.31 | 0.028 |
| QT interval (ms) | 40.4 ± 1.11 | 49.4 ± 2.23 | **0.001** | 45.3 ± 2.14 | 0.507 |
| QTc interval (ms) | 43.6 ± 0.98 | 48.9± 1.99 | 0.070 | 47.5 ± 1.86 | 0.999 |

HR: Heart rate; ISO: isoproterenol. The Kruskal-Wallis test followed by Dunn’s multiple comparison post-hoc test was performed to compare the WT vs RQ control (Con) or RQ control vs RQ gRNA groups. The bolded P values indicate statistical significance (P<0.05).

**Supplemental Table 2**. Ca^2+^ transient and spark parameters measured in isolated cells from WT and RQ treated mice with Ctr and gRNA.

| **Spark Parameters** | **WT** | **RQ-Con** | **P (WT vs. RQ-Con)** | **RQ-Edited** | **P (WT vs. RQ-gRNA)** | **P (RQ-Con vs. RQ-gRNA)** |
| --- | --- | --- | --- | --- | --- | --- |
| N (mice) | 5 | 6 |  | 6 |  |  |
| n (cells) | 20 | 20 |  | 17 |  |  |
| CaSpF | 1.05 ± 0.21 | 8.02 ± 1.59 | **<0.001** | 2.16 ± 0.47 | 0.723 | **<0.001** |
| Ca^2+^ spark amplitude (F/F0) | 0.53 ± 0.07 | 0.99 ± 0.08 | **<0.003** | 1.19 ± 0.09 | **<0.001** | 0.273 |
| Spark FWHM (µm) | 0.99 ± 0.06 | 1.17 ± 0.07 | 0.498 | 0.88 ± 0.05 | 0.531 | 0.099 |
| Spark FDHM (ms) | 13.05 ± 1.09 | 15.84 ± 1.33 | 0.468 | 12.16 ± 0.70 | 0.835 | 0.217 |
| Spark Full Width (µm) | 1.60 ± 0.17 | 2.08 ± 0.19 | 0.444 | 1.74± 0.18 | 0.881 | 0.738 |
| Spark Full Duration (ms) | 20.75 ± 2.32 | 31.36 ± 3.97 | 0.107 | 23.20 ± 2.27 | 0.879 | 0.264 |
| Spark Tau (ms) | 17.22 ± 2.19 | 23.01 ± 2.27 | 0.542 | 24.88 ± 6.74 | 0.377 | 0.943 |
| Spark TtP (ms) | 9.70 ± 1.36 | 11.40 ± 1.23 | 0.758 | 9.09 ± 1.04 | 0.929 | 0.554 |

FDHM, full duration at half-maximum; FWHM, full width at half-maximum; Tau, decay rate; TtP, time to peak. Data presented as mean ± SEM. Statistics based on Nested, one-way ANOVA. The bolded P values indicate statistical significance (P<0.05).

**Supplemental Table 3**. Overview of echocardiography parameters.

| **4-months** | **WT** | **RQ-Con** | **P (WT vs. RQ-Con)** | **RQ-Edited** | **P (RQ-Con vs. RQ-gRNA)** |
| --- | --- | --- | --- | --- | --- |
| N (mice) | 9 | 7 |  | 7 |  |
| Heart Rate (bpm) | 528.4 ± 13.3 | 486.4 ± 18.8 | 0.205 | 470.3 ± 32.6 | 0.905 |
| LVESD (mm) | 2.36 ± 0.10 | 2.59 ± 0.08 | 0.181 | 2.47 ± 0.12 | 0.661 |
| LVEDD (mm) | 3.80 ± 0.09 | 3.96 ± 0.09 | 0.462 | 3.84 ± 0.12 | 0.692 |
| LVESV (uL) | 19.8 ± 2.09 | 24.7 ± 1.73 | 0.206 | 22.2 ± 2.65 | 0.709 |
| LVEDV (uL) | 62.4 ± 3.74 | 68.8 ± 3.84 | 0.480 | 63.9 ± 4.83 | 0.721 |
| SV (uL) | 42.6 ± 2.04 | 44.1 ± 2.38 | 0.884 | 41.8 ± 2.54 | 0.792 |
| LVEF (%) | 68.7 ± 1.70 | 64.16 ± 1.24 | 0.113 | 65.9 ± 1.98 | 0.752 |
| LVFS (%) | 38.0 ± 1.30 | 34.7 ± 0.91 | 0.134 | 35.8 ± 1.53 | 0.816 |
| LV Mass (mg) | 22.5 ± 1.21 | 21.4 ± 1.30 | 0.059 | 19.5 ± 1.47 | 0.051 |
| LV Mass Corrected (mg) | 91.3 ± 4.07 | 109.5 ± 5.70 | 0.059 | 87.7 ± 5.92 | 0.054 |
| CO (mL/min) | 73.1 ± 3.25 | 87.6 ± 4.56 | 0.807 | 70.3 ± 4.81 | 0.602 |
| LVAW;s (mm) | 0.74 ± 0.02 | 0.87 ± 0.03 | **0.016** | 0.75 ± 0.04 | 0.108 |
| LVAW;d (mm) | 0.67 ± 0.03 | 0.79 ± 0.05 | 0.156 | 0.71 ± 0.03 | 0.438 |
| LVPW;s (mm) | 0.97 ± 0.06 | 0.97 ± 0.04 | 0.988 | 0.91 ± 0.05 | 0.609 |
| LVPW;d (mm) | 0.74 ± 0.02 | 0.75 ± 0.05 | 0.980 | 0.64 ± 0.04 | 0.275 |

| **8-months** | **WT** | **RQ-Con** | **P (WT vs. RQ-Con)** | **RQ-Edited** | **P (RQ-Con vs. RQ-gRNA)** |
| --- | --- | --- | --- | --- | --- |
| N (mice) | 9 | 7 |  | 7 |  |
| Heart Rate (bpm) | 507.1 ± 15.2 | 541.3 ± 7.4 | 0.151 | 487.3 ± 24.0 | 0.147 |
| LVESD (mm) | 2.42 ± 0.09 | 2.5 ± 0.13 | 0.875 | 2.36 ± 0.09 | 0.691 |
| LVEDD (mm) | 3.88 ± 0.14 | 3.93 ± 0.17 | 0.966 | 3.72 ± 0.16 | 0.649 |
| LVESV (uL) | 21.0 ± 1.95 | 23.0 ± 2.99 | 0.844 | 19.7 ± 1.98 | 0.649 |
| LVEDV (uL) | 66.1 ± 5.48 | 68.6 ± 6.87 | 0.955 | 60.0 ± 6.16 | 0.630 |
| SV (uL) | 45.1 ± 3.70 | 45.5 ± 4.43 | 0.997 | 40.3 ± 4.48 | 0.691 |
| LVEF (%) | 68.3 ± 0.91 | 66.9 ± 1.77 | 0.758 | 67.0 ± 1.26 | 0.999 |
| LVFS (%) | 37.6 ± 0.72 | 36.6 ± 1.26 | 0.758 | 36.6 ± 1.01 | >0.999 |
| LV Mass (mg) | 22.5 ± 1.35 | 24.7 ± 2.52 | 0.965 | 19.9 ± 2.80 | >0.999 |
| LV Mass Corrected (mg) | 109.5 ± 9.73 | 113.2 ± 11.2 | 0.966 | 113.3 ± 11.7 | >0.999 |
| CO (mL/min) | 87.6 ± 7.78 | 90.6 ± 8.98 | 0.730 | 90.7 ± 9.34 | 0.435 |
| LVAW;s (mm) | 0.79 ± 0.04 | 0.89 ± 0.07 | 0.424 | 0.84 ± 0.05 | 0.823 |
| LVAW;d (mm) | 0.80 ± 0.03 | 0.82 ± 0.05 | 0.917 | 0.80 ± 0.04 | 0.951 |
| LVPW;s (mm) | 0.97 ± 0.05 | 0.96 ± 0.04 | 0.986 | 0.99 ± 0.04 | 0.791 |
| LVPW;d (mm) | 0.76 ± 0.05 | 0.75 ± 0.05 | 0.988 | 0.87 ± 0.06 | 0.271 |

| **12-months** | **WT** | **RQ-Con** | **P (WT vs. RQ-Con)** | **RQ-Edited** | **P (RQ-Con vs. RQ-gRNA)** |
| --- | --- | --- | --- | --- | --- |
| N (mice) | 9 | 7 |  | 7 |  |
| Heart Rate (bpm) | 500.8 ± 16.3 | 502.3 ± 14.9 | 0.997 | 514.4 ± 9.7 | 0.778 |
| LVESD (mm) | 2.33 ± 0.15 | 2.58 ± 0.13 | 0.440 | 2.33 ± 0.11 | 0.370 |
| LVEDD (mm) | 3.68 ± 0.18 | 4.02 ± 0.16 | 0.358 | 3.76 ± 0.20 | 0.587 |
| LVESV (uL) | 19.8 ± 3.18 | 24.8 ± 2.93 | 0.497 | 19.3 ± 2.19 | 0.327 |
| LVEDV (uL) | 59.1 ± 6.83 | 71.7 ± 6.61 | 0.406 | 62.1 ± 7.62 | 0.621 |
| SV (uL) | 39.3 ± 3.80 | 46.9 ± 4.23 | 0.402 | 42.8 ± 5.48 | 0.828 |
| LVEF (%) | 67.6 ± 1.58 | 65.9 ± 1.99 | 0.780 | 68.8 ± 0.63 | 0.393 |
| LVFS (%) | 36.9 ± 1.14 | 36.0 ± 1.47 | 0.875 | 37.8 ± 0.60 | 0.522 |
| LV Mass (mg) | 19.4 ± 1.66 | 23.4 ± 1.79 | 0.150 | 21.9 ± 2.60 | 0.611 |
| LV Mass Corrected (mg) | 100.8 ± 9.13 | 124.4 ± 7.58 | **0.015** | 110.9 ± 11.7 | 0.611 |
| CO (mL/min) | 69.5 ± 6.92 | 99.5 ± 6.06 | 0.273 | 88.8 ± 9.32 | 0.890 |
| LVAW;s (mm) | 0.75 ± 0.02 | 0.94 ± 0.05 | **0.027** | 1.02 ± 0.05 | 0.563 |
| LVAW;d (mm) | 0.78 ± 0.03 | 0.87 ± 0.06 | 0.496 | 0.83 ± 0.02 | 0.877 |
| LVPW;s (mm) | 1.00 ± 0.04 | 0.97 ± 0.03 | 0.882 | 0.98 ± 0.07 | >0.999 |
| LVPW;d (mm) | 0.78 ± 0.02 | 0.79 ± 0.02 | 0.926 | 0.79 ± 0.04 | 1.000 |

Bpm, beats per minute; LVESD, left ventricular end-systolic diameter; LVEDD, left ventricular end-diastolic diameter; LVESV, left ventricular end-systolic volume; LVEDV, left ventricular end-diastolic volume; SV, stroke volume; LVEF, left ventricular ejection fraction; LVFS, left ventricular fractional shortening; CO, cardiac output; LVAW;s, left ventricular anterior wall diameter in systole; LVAW;d, left ventricular anterior wall diameter in diastole; LVPW;s, left ventricular posterior wall diameter in systole; LVPW;d, left ventricular posterior wall diameter in diastole. One-way ANOVA with Tukey’s post-hoc test was performed to compare the WT vs RQ control (Con) or RQ control vs RQ gRNA groups. The bolded P values indicate statistical significance (P<0.05).

**Supplemental Table 4. ECG parameters at baseline and after isoproterenol at 12 months post-AAV9.**

| **Baseline** | **WT** | **RQ-Con** | **P (WT vs. RQ-Con)** | **RQ-Edited** | **P (RQ-Con vs. RQ-gRNA)** |
| --- | --- | --- | --- | --- | --- |
| N (mice) | 6 | 7 |  | 5 |  |
| HR (bpm) | 550.8± 5.9 | 503.1± 15.2 | **0.040** | 524.6 ± 16.8 | 0.977 |
| P wave (ms) | 16.1 ± 0.82 | 17.1± 1.11 | 0.999 | 18.1 ± 0.67 | 0.638 |
| PR interval (ms) | 49.2 ± 2.52 | 44.8 ± 1.61 | 0.490 | 50.7 ± 1.86 | 0.119 |
| QRS interval (ms) | 20.5 ± 0.98 | 21.8 ± 1.05 | 0.912 | 24.6 ± 0.92 | 0.159 |
| QT interval (ms) | 49.0 ± 1.13 | 49.9 ± 2.38 | 0.999 | 49.2 ± 1.98 | 0.999 |
| QTc interval (ms) | 45.9 ± 1.85 | 46.7 ± 2.21 | 0.999 | 49.9 ± 2.94 | 0.999 |

| **ISO + Caffeine** | **WT** | **RQ-Con** | **P (WT vs. RQ-Con)** | **RQ-Edited** | **P (RQ-Con vs. RQ-gRNA)** |
| --- | --- | --- | --- | --- | --- |
| N (mice) | 6 | 7 |  | 5 |  |
| HR (bpm) | 640.2 ± 9.9 | 610.4± 14.9 | 0.269 | 668.8 ± 9.8 | **0.012** |
| P wave (ms) | 15.1 ± 0.98 | 15.1 ± 1.10 | 0.999 | 15.6 ± 1.33 | 0.999 |
| PR interval (ms) | 24.3 ± 4.67 | 27.2 ± 4.40 | 0.999 | 29.0 ± 5.63 | 0.999 |
| QRS interval (ms) | 20.0 ± 0.90 | 22.5 ± 1.55 | 0.847 | 22.1 ± 2.61 | 0.999 |
| QT interval (ms) | 44.7 ± 1.82 | 49.5 ± 2.39 | 0.847 | 46.7 ± 2.38 | 0.904 |
| QTc interval (ms) | 46.7 ± 0.66 | 48.6 ± 2.07 | 0.999 | 45.9 ± 1.25 | 0.998 |

HR: Heart rate; ISO: isoproterenol. The Kruskal-Wallis test followed by Dunn’s multiple comparison post-hoc test was performed to compare the WT vs RQ control (Con) or RQ control vs RQ gRNA groups. The bolded P values indicate statistical significance (P<0.05).

**Supplemental Figure 1.** Sequence of CRISPR/Cas9 genome editing and control plasmids used to generate adeno-associated viral vectors.

**A. pAAV-U6-BbsI-gRNA-TNT-SACas9-HA-OLLAS-spA (control plasmid)**

Legend: ITR, U6 Promoter, BbsI cloning site, Guide RNA Scaffold, Human Troponin T Promoter, NLS, SaCas9

cagctgcgcgctcgctcgctcactgaggccgcccgggcaaagcccgggcgtcgggcgacctttggtcgcccggcctcagtgagcgagcgagcgcgcagagagggagtggccaactccatcactaggggttccttgtagttaatgattaacccgccatgctacttatctacgtagccatgctctggTGTACAAAAAAGCAGGCTTTAAAGGAACCAATTCAGTCGACTGGATCCGGTACCAAGGTCGGGCAGGAAGAGGGCCTATTTCCCATGATTCCTTCATATTTGCATATACGATACAAGGCTGTTAGAGAGATAATTAGAATTAATTTGACTGTAAACACAAAGATATTAGTACAAAATACGTGACGTAGAAAGTAATAATTTCTTGGGTAGTTTGCAGTTTTAAAATTATGTTTTAAAATGGACTATCATATGCTTACCGTAACTTGAAAGTATTTCGATTTCTTGGCTTTATATATCTTGTGGAAAGGACGAAACACCgggtcttcacgcgtgaagacCagttttagtactctggaaacagaatctactaaaacaaggcaaaatgccgtgtttatctcgtcaacttgttggcgagattttttTCTAGACTCAGTCCATTAGGAGCCAGTAGCCTGGAAGATGTCTTTACCCCCAGCATCAGTTCAAGTGGAGCAGCACATAACTCTTGCCCTCTGCCTTCCAAGATTCTGGTGCTGAGACTTATGGAGTGTCTTGGAGGTTGCCTTCTGCCCCCCAACCCTGCTCCCAGCTGGCCCTCCCAGGCCTGGGTTGCTGGCCTCTGCTTTATCAGGATTCTCAAGAGGGACAGCTGGTTTATGTTGCATGACTGTTCCCTGCATATCTGCTCTGGTTTTAAATAGCTTATCTGAGCAGCTGGAGGACCACATGGGCTTATATGGCGTGGGGTACATGTTCCTGTAGCCTTGTCCCTGGCACCTGCCAAAATAGCAGCCAACACCCCCCACCCCCACCGCCATCCCCCTGCCCCACCCGTCCCCTGTCGCACATTCCTCCCTCCGCAGGGCTGGCTCACCAGGCCCCAGCCCACATGCCTGCTTAAAGCCCTCTCCATCCTCTGCCTCACCCAGTCCCCGCTGAGACTGAGCAGACGCCTCCAGGATCTGTCGGCAGccATGGCCCCAAAGAAGAAGCGGAAGGTCGGTATCCACGGAGTCCCAGCAGCCAAGCGGAACTACATCCTGGGCCTGGACATCGGCATCACCAGCGTGGGCTACGGCATCATCGACTACGAGACACGGGACGTGATCGATGCCGGCGTGCGGCTGTTCAAAGAGGCCAACGTGGAAAACAACGAGGGCAGGCGGAGCAAGAGAGGCGCCAGAAGGCTGAAGCGGCGGAGGCGGCATAGAATCCAGAGAGTGAAGAAGCTGCTGTTCGACTACAACCTGCTGACCGACCACAGCGAGCTGAGCGGCATCAACCCCTACGAGGCCAGAGTGAAGGGCCTGAGCCAGAAGCTGAGCGAGGAAGAGTTCTCTGCCGCCCTGCTGCACCTGGCCAAGAGAAGAGGCGTGCACAACGTGAACGAGGTGGAAGAGGACACCGGCAACGAGCTGTCCACCAAAGAGCAGATCAGCCGGAACAGCAAGGCCCTGGAAGAGAAATACGTGGCCGAACTGCAGCTGGAACGGCTGAAGAAAGACGGCGAAGTGCGGGGCAGCATCAACAGATTCAAGACCAGCGACTACGTGAAAGAAGCCAAACAGCTGCTGAAGGTGCAGAAGGCCTACCACCAGCTGGACCAGAGCTTCATCGACACCTACATCGACCTGCTGGAAACCCGGCGGACCTACTATGAGGGACCTGGCGAGGGCAGCCCCTTCGGCTGGAAGGACATCAAAGAATGGTACGAGATGCTGATGGGCCACTGCACCTACTTCCCCGAGGAACTGCGGAGCGTGAAGTACGCCTACAACGCCGACCTGTACAACGCCCTGAACGACCTGAACAATCTCGTGATCACCAGGGACGAGAACGAGAAGCTGGAATATTACGAGAAGTTCCAGATCATCGAGAACGTGTTCAAGCAGAAGAAGAAGCCCACCCTGAAGCAGATCGCCAAAGAAATCCTCGTGAACGAAGAGGATATTAAGGGCTACAGAGTGACCAGCACCGGCAAGCCCGAGTTCACCAACCTGAAGGTGTACCACGACATCAAGGACATTACCGCCCGGAAAGAGATTATTGAGAACGCCGAGCTGCTGGATCAGATTGCCAAGATCCTGACCATCTACCAGAGCAGCGAGGACATCCAGGAAGAACTGACCAATCTGAACTCCGAGCTGACCCAGGAAGAGATCGAGCAGATCTCTAATCTGAAGGGCTATACCGGCACCCACAACCTGAGCCTGAAGGCCATCAACCTGATCCTGGACGAGCTGTGGCACACCAACGACAACCAGATCGCTATCTTCAACCGGCTGAAGCTGGTGCCCAAGAAGGTGGACCTGTCCCAGCAGAAAGAGATCCCCACCACCCTGGTGGACGACTTCATCCTGAGCCCCGTCGTGAAGAGAAGCTTCATCCAGAGCATCAAAGTGATCAACGCCATCATCAAGAAGTACGGCCTGCCCAACGACATCATTATCGAGCTGGCCCGCGAGAAGAACTCCAAGGACGCCCAGAAAATGATCAACGAGATGCAGAAGCGGAACCGGCAGACCAACGAGCGGATCGAGGAAATCATCCGGACCACCGGCAAAGAGAACGCCAAGTACCTGATCGAGAAGATCAAGCTGCACGACATGCAGGAAGGCAAGTGCCTGTACAGCCTGGAAGCCATCCCTCTGGAAGATCTGCTGAACAACCCCTTCAACTATGAGGTGGACCACATCATCCCCAGAAGCGTGTCCTTCGACAACAGCTTCAACAACAAGGTGCTCGTGAAGCAGGAAGAAAACAGCAAGAAGGGCAACCGGACCCCATTCCAGTACCTGAGCAGCAGCGACAGCAAGATCAGCTACGAAACCTTCAAGAAGCACATCCTGAATCTGGCCAAGGGCAAGGGCAGAATCAGCAAGACCAAGAAAGAGTATCTGCTGGAAGAACGGGACATCAACAGGTTCTCCGTGCAGAAAGACTTCATCAACCGGAACCTGGTGGATACCAGATACGCCACCAGAGGCCTGATGAACCTGCTGCGGAGCTACTTCAGAGTGAACAACCTGGACGTGAAAGTGAAGTCCATCAATGGCGGCTTCACCAGCTTTCTGCGGCGGAAGTGGAAGTTTAAGAAAGAGCGGAACAAGGGGTACAAGCACCACGCCGAGGACGCCCTGATCATTGCCAACGCCGATTTCATCTTCAAAGAGTGGAAGAAACTGGACAAGGCCAAAAAAGTGATGGAAAACCAGATGTTCGAGGAAAAGCAGGCCGAGAGCATGCCCGAGATCGAAACCGAGCAGGAGTACAAAGAGATCTTCATCACCCCCCACCAGATCAAGCACATTAAGGACTTCAAGGACTACAAGTACAGCCACCGGGTGGACAAGAAGCCTAATAGAGAGCTGATTAACGACACCCTGTACTCCACCCGGAAGGACGACAAGGGCAACACCCTGATCGTGAACAATCTGAACGGCCTGTACGACAAGGACAATGACAAGCTGAAAAAGCTGATCAACAAGAGCCCCGAAAAGCTGCTGATGTACCACCACGACCCCCAGACCTACCAGAAACTGAAGCTGATTATGGAACAGTACGGCGACGAGAAGAATCCCCTGTACAAGTACTACGAGGAAACCGGGAACTACCTGACCAAGTACTCCAAAAAGGACAACGGCCCCGTGATCAAGAAGATTAAGTATTACGGCAACAAACTGAACGCCCATCTGGACATCACCGACGACTACCCCAACAGCAGAAACAAGGTCGTGAAGCTGTCCCTGAAGCCCTACAGATTCGACGTGTACCTGGACAATGGCGTGTACAAGTTCGTGACCGTGAAGAATCTGGATGTGATCAAAAAAGAAAACTACTACGAAGTGAATAGCAAGTGCTATGAGGAAGCTAAGAAGCTGAAGAAGATCAGCAACCAGGCCGAGTTTATCGCCTCCTTCTACAACAACGATCTGATCAAGATCAACGGCGAGCTGTATAGAGTGATCGGCGTGAACAACGACCTGCTGAACCGGATCGAAGTGAACATGATCGACATCACCTACCGCGAGTACCTGGAAAACATGAACGACAAGAGGCCCCCCAGGATCATTAAGACAATCGCCTCCAAGACCCAGAGCATTAAGAAGTACAGCACAGACATTCTGGGCAACCTGTATGAAGTGAAATCTAAGAAGCACCCTCAGATCATCAAAAAGGGCAAAAGGCCGGCGGCCACGAAAAAGGCCGGCCAGGCAAAAAAGAAAAAGggatcctacccatacgatgttccagattacgctagcggcttcgccaacgagcttggacccaggttgatgggaaagtaagaattcctagagcgaataaaagatctttattttcattagatctgtgtgttggttttttgtgtgatgcagctacgtagataagtagcatggcgggttaatcattaactacaaggaacccctagtgatggagttggccactccctctctgcgcgctcgctcgctcactgaggccgggcgaccaaaggtcgcccgacgcccgggctttgcccgggcggcctcagtgagcgagcgagcgcgcagctgcattaatgaatcggccaacgcgcggggagaggcggtttgcgtattgggcgctcttccgcttcctcgctcactgactcgctgcgctcggtcgttcggctgcggcgagcggtatcagctcactcaaaggcggtaatacggttatccacagaatcaggggataacgcaggaaagaacatgtgagcaaaaccgcagcaaaaggccaggaaccgtaaaaaggccgcgttgctggcgtttttccataggctccgcccccctgacgagcatcacaaaaatcgacgctcaagtcagaggtggcgaaacccgacaggactataaagataccaggcgtttccccctggaagctccctcgtgcgctctcctgttccgaccctgccgcttaccggatacctgtccgcctttctcccttcgggaagcgtggcgctttctcatagctcacgctgtaggtatctcagttcggtgtaggtcgttcgctccaagctgggctgtgtgcacgaaccccccgttcagcccgaccgctgcgccttatccggtaactatcgtcttgagtccaacccggtaagacacgacttatcgccactggcagcagccactggtaacaggattagcagagcgaggtatgtaggcggtgctacagagttcttgaagtggtggcctaactacggctacactagaaggacagtatttggtatctgcgctctgctgaagccagttaccttcggaaaaagagttggtagctcttgatccggcaaacaaaccaccgctggtagcggtggtttttttgtttgcaagcagcagattacgcgcagaaaaaaaggatctcaagaagatcctttgatcttttctacggggtctgacgctcagtggaacgaaaactcacgttaagggattttggtcatgagattatcaaaaaggatcttcacctagatccttttaaattaaaaatgaagttttaaatcaatctaaagtatatatgagtaaacttggtctgacagttaccaatgcttaatcagtgaggcacctatctcagcgatctgtctatttcgttcatccatagttgcctgactccccgtcgtgtagataactacgatacgggagggcttaccatctggccccagtgctgcaatgataccgcgagacccacgctcaccggctccagatttatcagcaataaaccagccagccggaagggccgagcgcagaagtggtcctgcaactttatccgcctccatccagtctattaattgttgccgggaagctagagtaagtagttcgccagttaatagtttgcgcaacgttgttaccattactacaggcatcgtggtgtcacgctcgtcgtttggtatggcttcattcagctccggttcccaacgatcaaggcgagttacatgatcccccatgttgtgcaaaaaagcggttagctccttcggtcctccgatcgttgtcagaagtaagttggccgcagtgttatcactcatggttatggcagcactgcataattctcttactgtcatgccatccgtaagatgcttttctgtgactggtgagtactcaaccaagtcattctgagaatagtgtatgcggcgaccgagttgctcttgcccggcgtcaatacgggataataccgcgccacatagcagaactttaaaagtgctcatcattggaaaacgttcttcggggcgaaaactctcaaggatcttaccactattgagatccagttcgatgtaacccactcgtgcacccaactgatcttcagcatcttttactttcaccagcgtttctgggtgagcaaaaacaggaaggcaaaatgccgcaaaaaagggaataagggcgacacggaaatgttgaatactcatactcttcctttttcaatattattgaagcatttatcagggttattgtctcatgagcggatacatatttgaatgtatttagaaaaataaacaaataggggttccgcgcacatttccccgaaagatgccacctgaaattataaacgttaatattttgttaaaattcgcgttaaatttttgttaaatcagctcattttttaaccaataggccgaaatcggaaaaatcccttataaatcaaaagaatagaccgagatagggttgagtgttgttccagtttggaacaagagtccactattgaggaacgtgaactccagcgtcaaagggcgaaaaaccgtctatcggggcgatggcccactacgtgaaccatcaccctaatcaagttttttggggtcgaggtgccgtaaagcactaaatcggaaccctaaagggagcccccgatttagagcttgacggggaaagccggcgaacgtggcgagaaaggaagggaagaaagcgaaaggagcgggcgctagggcgctggcaagtgtagcggtcacgctgcgcgtaaccaccacacccgccgcgcttaatgcgccgctacagggcgcgtcccattcgccattcaggctgcgcaactgttgggaagggcgatcggtgcgggcctcttcgctattacgc

**B. pAAV-U6-gRNA-TNT-SACas9-HA-OLLAS-spA (gRNA plasmid)**

ITR

U6 Promoter

Guide RNA

Human Troponin T Promoter

NLS

SaCas9

cagctgcgcgctcgctcgctcactgaggccgcccgggcaaagcccgggcgtcgggcgacctttggtcgcccggcctcagtgagcgagcgagcgcgcagagagggagtggccaactccatcactaggggttccttgtagttaatgattaacccgccatgctacttatctacgtagccatgctctggTGTACAAAAAAGCAGGCTTTAAAGGAACCAATTCAGTCGACTGGATCCGGTACCAAGGTCGGGCAGGAAGAGGGCCTATTTCCCATGATTCCTTCATATTTGCATATACGATACAAGGCTGTTAGAGAGATAATTAGAATTAATTTGACTGTAAACACAAAGATATTAGTACAAAATACGTGACGTAGAAAGTAATAATTTCTTGGGTAGTTTGCAGTTTTAAAATTATGTTTTAAAATGGACTATCATATGCTTACCGTAACTTGAAAGTATTTCGATTTCTTGGCTTTATATATCTTGTGGAAAGGACGAAACACCgCCGAAGGAGAAAAAGTGCAAGgttttagtactctggaaacagaatctactaaaacaaggcaaaatgccgtgtttatctcgtcaacttgttggcgagattttttTCTAGACTCAGTCCATTAGGAGCCAGTAGCCTGGAAGATGTCTTTACCCCCAGCATCAGTTCAAGTGGAGCAGCACATAACTCTTGCCCTCTGCCTTCCAAGATTCTGGTGCTGAGACTTATGGAGTGTCTTGGAGGTTGCCTTCTGCCCCCCAACCCTGCTCCCAGCTGGCCCTCCCAGGCCTGGGTTGCTGGCCTCTGCTTTATCAGGATTCTCAAGAGGGACAGCTGGTTTATGTTGCATGACTGTTCCCTGCATATCTGCTCTGGTTTTAAATAGCTTATCTGAGCAGCTGGAGGACCACATGGGCTTATATGGCGTGGGGTACATGTTCCTGTAGCCTTGTCCCTGGCACCTGCCAAAATAGCAGCCAACACCCCCCACCCCCACCGCCATCCCCCTGCCCCACCCGTCCCCTGTCGCACATTCCTCCCTCCGCAGGGCTGGCTCACCAGGCCCCAGCCCACATGCCTGCTTAAAGCCCTCTCCATCCTCTGCCTCACCCAGTCCCCGCTGAGACTGAGCAGACGCCTCCAGGATCTGTCGGCAGccATGGCCCCAAAGAAGAAGCGGAAGGTCGGTATCCACGGAGTCCCAGCAGCCAAGCGGAACTACATCCTGGGCCTGGACATCGGCATCACCAGCGTGGGCTACGGCATCATCGACTACGAGACACGGGACGTGATCGATGCCGGCGTGCGGCTGTTCAAAGAGGCCAACGTGGAAAACAACGAGGGCAGGCGGAGCAAGAGAGGCGCCAGAAGGCTGAAGCGGCGGAGGCGGCATAGAATCCAGAGAGTGAAGAAGCTGCTGTTCGACTACAACCTGCTGACCGACCACAGCGAGCTGAGCGGCATCAACCCCTACGAGGCCAGAGTGAAGGGCCTGAGCCAGAAGCTGAGCGAGGAAGAGTTCTCTGCCGCCCTGCTGCACCTGGCCAAGAGAAGAGGCGTGCACAACGTGAACGAGGTGGAAGAGGACACCGGCAACGAGCTGTCCACCAAAGAGCAGATCAGCCGGAACAGCAAGGCCCTGGAAGAGAAATACGTGGCCGAACTGCAGCTGGAACGGCTGAAGAAAGACGGCGAAGTGCGGGGCAGCATCAACAGATTCAAGACCAGCGACTACGTGAAAGAAGCCAAACAGCTGCTGAAGGTGCAGAAGGCCTACCACCAGCTGGACCAGAGCTTCATCGACACCTACATCGACCTGCTGGAAACCCGGCGGACCTACTATGAGGGACCTGGCGAGGGCAGCCCCTTCGGCTGGAAGGACATCAAAGAATGGTACGAGATGCTGATGGGCCACTGCACCTACTTCCCCGAGGAACTGCGGAGCGTGAAGTACGCCTACAACGCCGACCTGTACAACGCCCTGAACGACCTGAACAATCTCGTGATCACCAGGGACGAGAACGAGAAGCTGGAATATTACGAGAAGTTCCAGATCATCGAGAACGTGTTCAAGCAGAAGAAGAAGCCCACCCTGAAGCAGATCGCCAAAGAAATCCTCGTGAACGAAGAGGATATTAAGGGCTACAGAGTGACCAGCACCGGCAAGCCCGAGTTCACCAACCTGAAGGTGTACCACGACATCAAGGACATTACCGCCCGGAAAGAGATTATTGAGAACGCCGAGCTGCTGGATCAGATTGCCAAGATCCTGACCATCTACCAGAGCAGCGAGGACATCCAGGAAGAACTGACCAATCTGAACTCCGAGCTGACCCAGGAAGAGATCGAGCAGATCTCTAATCTGAAGGGCTATACCGGCACCCACAACCTGAGCCTGAAGGCCATCAACCTGATCCTGGACGAGCTGTGGCACACCAACGACAACCAGATCGCTATCTTCAACCGGCTGAAGCTGGTGCCCAAGAAGGTGGACCTGTCCCAGCAGAAAGAGATCCCCACCACCCTGGTGGACGACTTCATCCTGAGCCCCGTCGTGAAGAGAAGCTTCATCCAGAGCATCAAAGTGATCAACGCCATCATCAAGAAGTACGGCCTGCCCAACGACATCATTATCGAGCTGGCCCGCGAGAAGAACTCCAAGGACGCCCAGAAAATGATCAACGAGATGCAGAAGCGGAACCGGCAGACCAACGAGCGGATCGAGGAAATCATCCGGACCACCGGCAAAGAGAACGCCAAGTACCTGATCGAGAAGATCAAGCTGCACGACATGCAGGAAGGCAAGTGCCTGTACAGCCTGGAAGCCATCCCTCTGGAAGATCTGCTGAACAACCCCTTCAACTATGAGGTGGACCACATCATCCCCAGAAGCGTGTCCTTCGACAACAGCTTCAACAACAAGGTGCTCGTGAAGCAGGAAGAAAACAGCAAGAAGGGCAACCGGACCCCATTCCAGTACCTGAGCAGCAGCGACAGCAAGATCAGCTACGAAACCTTCAAGAAGCACATCCTGAATCTGGCCAAGGGCAAGGGCAGAATCAGCAAGACCAAGAAAGAGTATCTGCTGGAAGAACGGGACATCAACAGGTTCTCCGTGCAGAAAGACTTCATCAACCGGAACCTGGTGGATACCAGATACGCCACCAGAGGCCTGATGAACCTGCTGCGGAGCTACTTCAGAGTGAACAACCTGGACGTGAAAGTGAAGTCCATCAATGGCGGCTTCACCAGCTTTCTGCGGCGGAAGTGGAAGTTTAAGAAAGAGCGGAACAAGGGGTACAAGCACCACGCCGAGGACGCCCTGATCATTGCCAACGCCGATTTCATCTTCAAAGAGTGGAAGAAACTGGACAAGGCCAAAAAAGTGATGGAAAACCAGATGTTCGAGGAAAAGCAGGCCGAGAGCATGCCCGAGATCGAAACCGAGCAGGAGTACAAAGAGATCTTCATCACCCCCCACCAGATCAAGCACATTAAGGACTTCAAGGACTACAAGTACAGCCACCGGGTGGACAAGAAGCCTAATAGAGAGCTGATTAACGACACCCTGTACTCCACCCGGAAGGACGACAAGGGCAACACCCTGATCGTGAACAATCTGAACGGCCTGTACGACAAGGACAATGACAAGCTGAAAAAGCTGATCAACAAGAGCCCCGAAAAGCTGCTGATGTACCACCACGACCCCCAGACCTACCAGAAACTGAAGCTGATTATGGAACAGTACGGCGACGAGAAGAATCCCCTGTACAAGTACTACGAGGAAACCGGGAACTACCTGACCAAGTACTCCAAAAAGGACAACGGCCCCGTGATCAAGAAGATTAAGTATTACGGCAACAAACTGAACGCCCATCTGGACATCACCGACGACTACCCCAACAGCAGAAACAAGGTCGTGAAGCTGTCCCTGAAGCCCTACAGATTCGACGTGTACCTGGACAATGGCGTGTACAAGTTCGTGACCGTGAAGAATCTGGATGTGATCAAAAAAGAAAACTACTACGAAGTGAATAGCAAGTGCTATGAGGAAGCTAAGAAGCTGAAGAAGATCAGCAACCAGGCCGAGTTTATCGCCTCCTTCTACAACAACGATCTGATCAAGATCAACGGCGAGCTGTATAGAGTGATCGGCGTGAACAACGACCTGCTGAACCGGATCGAAGTGAACATGATCGACATCACCTACCGCGAGTACCTGGAAAACATGAACGACAAGAGGCCCCCCAGGATCATTAAGACAATCGCCTCCAAGACCCAGAGCATTAAGAAGTACAGCACAGACATTCTGGGCAACCTGTATGAAGTGAAATCTAAGAAGCACCCTCAGATCATCAAAAAGGGCAAAAGGCCGGCGGCCACGAAAAAGGCCGGCCAGGCAAAAAAGAAAAAGggatcctacccatacgatgttccagattacgctagcggcttcgccaacgagcttggacccaggttgatgggaaagtaagaattcctagagcgaataaaagatctttattttcattagatctgtgtgttggttttttgtgtgatgcagctacgtagataagtagcatggcgggttaatcattaactacaaggaacccctagtgatggagttggccactccctctctgcgcgctcgctcgctcactgaggccgggcgaccaaaggtcgcccgacgcccgggctttgcccgggcggcctcagtgagcgagcgagcgcgcagctgcattaatgaatcggccaacgcgcggggagaggcggtttgcgtattgggcgctcttccgcttcctcgctcactgactcgctgcgctcggtcgttcggctgcggcgagcggtatcagctcactcaaaggcggtaatacggttatccacagaatcaggggataacgcaggaaagaacatgtgagcaaaaccgcagcaaaaggccaggaaccgtaaaaaggccgcgttgctggcgtttttccataggctccgcccccctgacgagcatcacaaaaatcgacgctcaagtcagaggtggcgaaacccgacaggactataaagataccaggcgtttccccctggaagctccctcgtgcgctctcctgttccgaccctgccgcttaccggatacctgtccgcctttctcccttcgggaagcgtggcgctttctcatagctcacgctgtaggtatctcagttcggtgtaggtcgttcgctccaagctgggctgtgtgcacgaaccccccgttcagcccgaccgctgcgccttatccggtaactatcgtcttgagtccaacccggtaagacacgacttatcgccactggcagcagccactggtaacaggattagcagagcgaggtatgtaggcggtgctacagagttcttgaagtggtggcctaactacggctacactagaaggacagtatttggtatctgcgctctgctgaagccagttaccttcggaaaaagagttggtagctcttgatccggcaaacaaaccaccgctggtagcggtggtttttttgtttgcaagcagcagattacgcgcagaaaaaaaggatctcaagaagatcctttgatcttttctacggggtctgacgctcagtggaacgaaaactcacgttaagggattttggtcatgagattatcaaaaaggatcttcacctagatccttttaaattaaaaatgaagttttaaatcaatctaaagtatatatgagtaaacttggtctgacagttaccaatgcttaatcagtgaggcacctatctcagcgatctgtctatttcgttcatccatagttgcctgactccccgtcgtgtagataactacgatacgggagggcttaccatctggccccagtgctgcaatgataccgcgagacccacgctcaccggctccagatttatcagcaataaaccagccagccggaagggccgagcgcagaagtggtcctgcaactttatccgcctccatccagtctattaattgttgccgggaagctagagtaagtagttcgccagttaatagtttgcgcaacgttgttaccattactacaggcatcgtggtgtcacgctcgtcgtttggtatggcttcattcagctccggttcccaacgatcaaggcgagttacatgatcccccatgttgtgcaaaaaagcggttagctccttcggtcctccgatcgttgtcagaagtaagttggccgcagtgttatcactcatggttatggcagcactgcataattctcttactgtcatgccatccgtaagatgcttttctgtgactggtgagtactcaaccaagtcattctgagaatagtgtatgcggcgaccgagttgctcttgcccggcgtcaatacgggataataccgcgccacatagcagaactttaaaagtgctcatcattggaaaacgttcttcggggcgaaaactctcaaggatcttaccactattgagatccagttcgatgtaacccactcgtgcacccaactgatcttcagcatcttttactttcaccagcgtttctgggtgagcaaaaacaggaaggcaaaatgccgcaaaaaagggaataagggcgacacggaaatgttgaatactcatactcttcctttttcaatattattgaagcatttatcagggttattgtctcatgagcggatacatatttgaatgtatttagaaaaataaacaaataggggttccgcgcacatttccccgaaagatgccacctgaaattataaacgttaatattttgttaaaattcgcgttaaatttttgttaaatcagctcattttttaaccaataggccgaaatcggaaaaatcccttataaatcaaaagaatagaccgagatagggttgagtgttgttccagtttggaacaagagtccactattgaggaacgtgaactccagcgtcaaagggcgaaaaaccgtctatcggggcgatggcccactacgtgaaccatcaccctaatcaagttttttggggtcgaggtgccgtaaagcactaaatcggaaccctaaagggagcccccgatttagagcttgacggggaaagccggcgaacgtggcgagaaaggaagggaagaaagcgaaaggagcgggcgctagggcgctggcaagtgtagcggtcacgctgcgcgtaaccaccacacccgccgcgcttaatgcgccgctacagggcgcgtcccattcgccattcaggctgcgcaactgttgggaagggcgatcggtgcgggcctcttcgctattacgc

**Supplemental Figure 2**. Baseline ECG tracings and intracardiac ventricular electrograms obtained in mice 6-weeks after AAV9 injections.


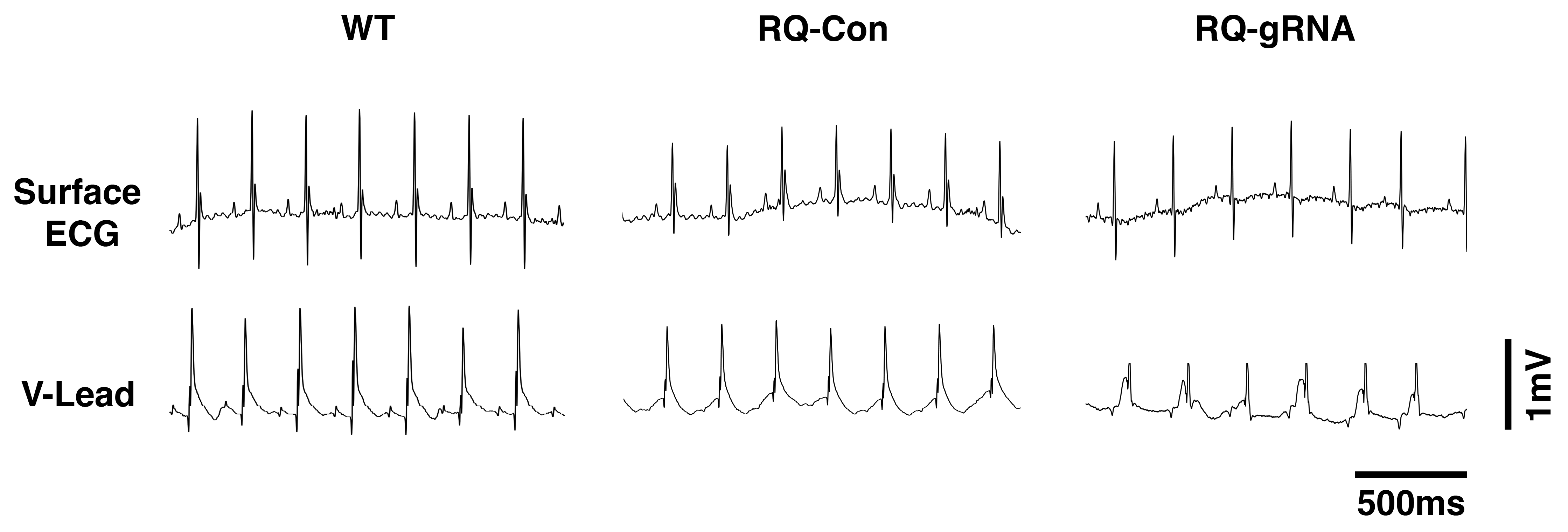


**Supplemental Figure 3.** Baseline ECG tracings and intracardiac ventricular electrograms obtained in mice 12-months after AAV9 injections. * indicates amplifier artefact.


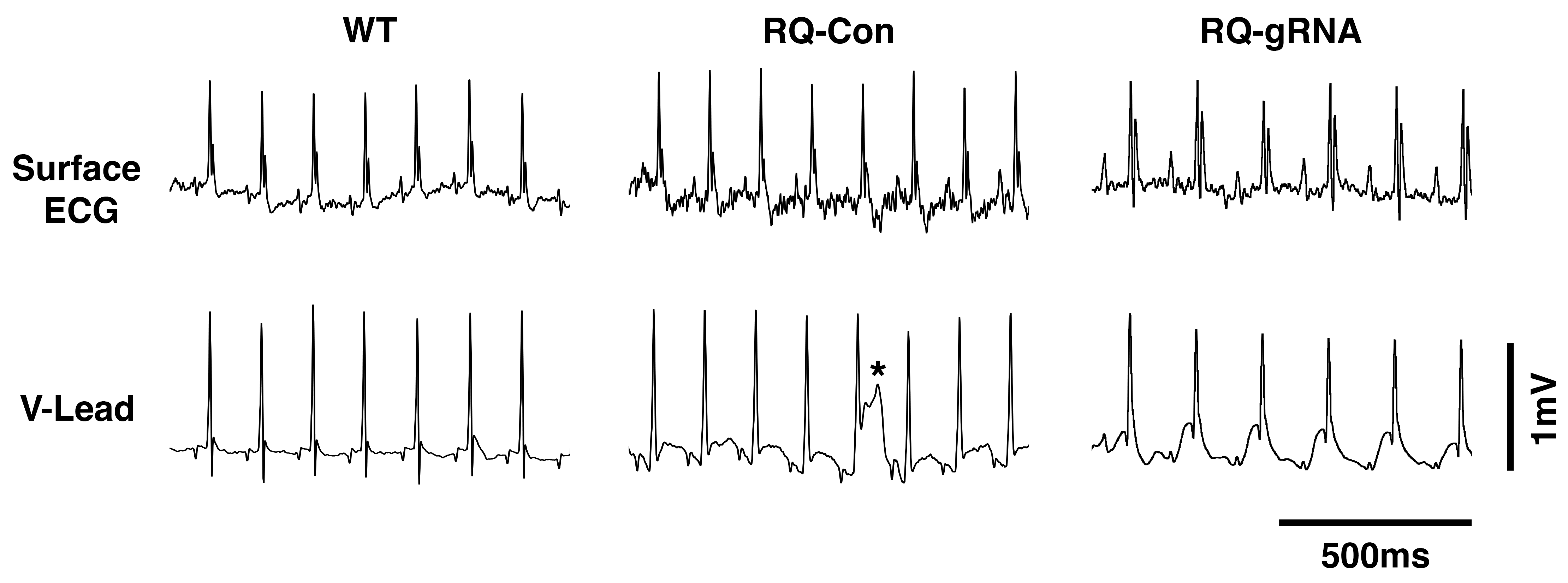


**Supplemental Figure 4. H&E Staining of Heart and Liver Sections after Long-Term Genome Editing.** (**A**) Hematoxylin and eosin (H&E) staining of transverse cardiac sections showing normal cardiac structure and dimensions at 1x magnification and normal tissue organization. (**B**) H&E staining of liver sections showing normal tissue structure and absence of immune cell infiltration.


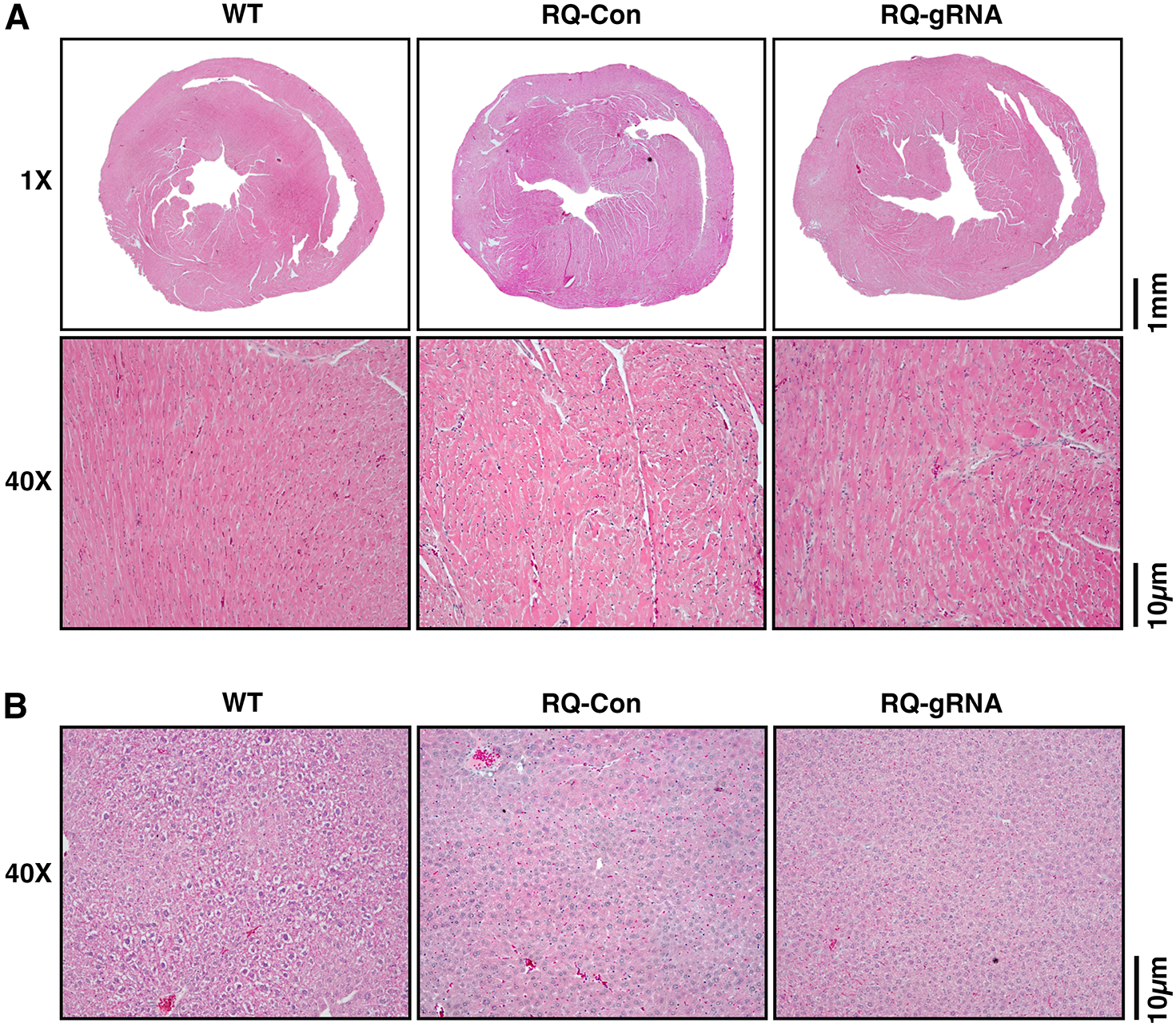


**Supplemental Figure 5. Persistent SaCas9 expression after genome editing.**(**A**) Schematic of AAV genome with U6 genomic DNA (gDNA) and SaCas9 cDNA primers shown below. (**B**) PCR amplification of AAV genome in ventricles of R176Q/+ mice at 12 months after CRISPR/Cas9 AAV9 injection (RQ-gRNA) compared with non-injected control mice (RQ-Con). (**C**) cDNA qRT-PCR quantification of SaCas9 mRNA levels relative to GAPDH mRNA.  Data is shown as mean ± SEM. After confirming normality, unpaired student t-test was performed.

**
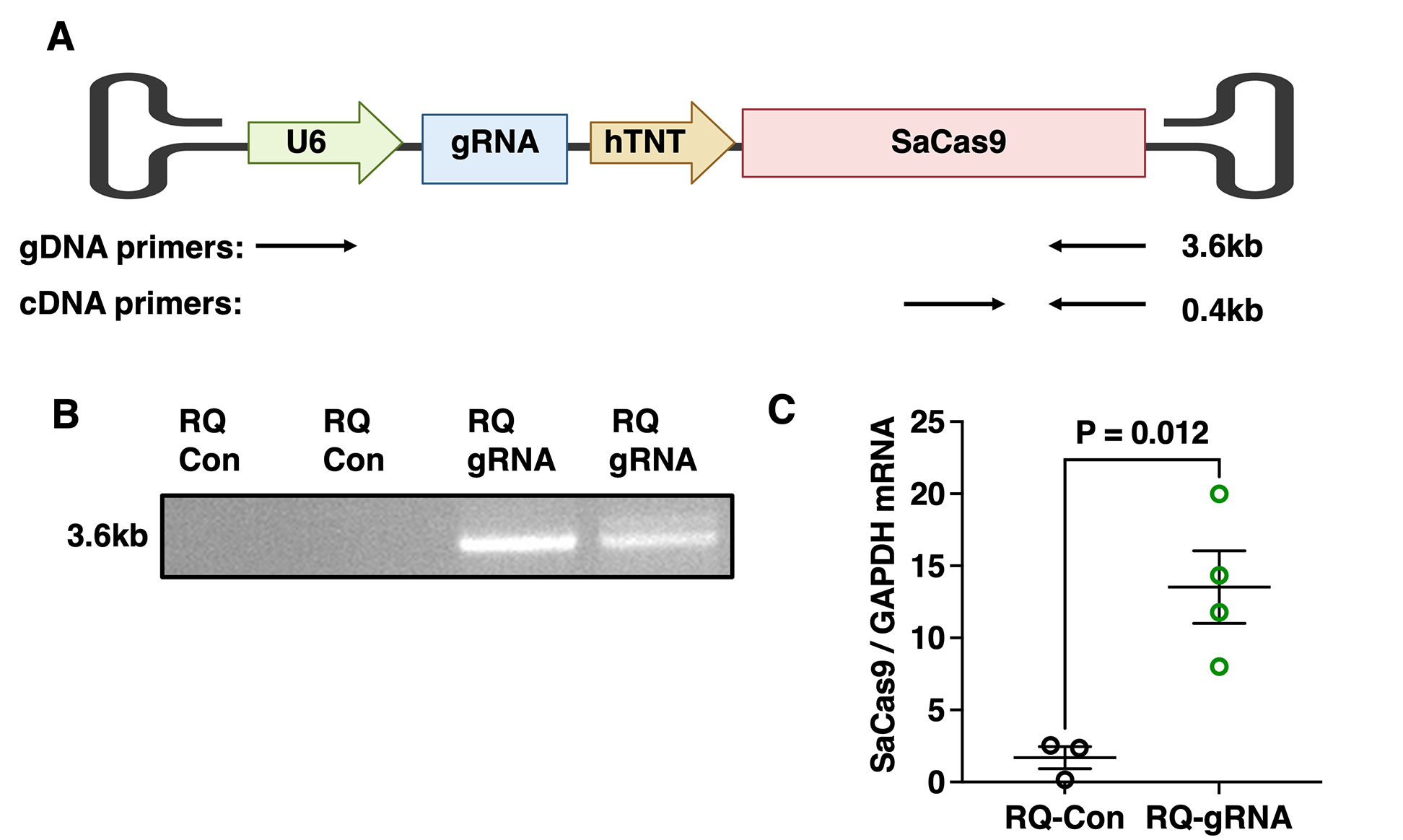
**

**Supplemental References**

1. Xiao X, Li J, Samulski RJ. Production of high-titer recombinant adeno-associated virus vectors in the absence of helper adenovirus. *J Virol* 1998;72:2224-32.10.1128/JVI.72.3.2224-2232.1998.

2. Pan X, Philippen L, Lahiri SK, et al. In Vivo Ryr2 Editing Corrects Catecholaminergic Polymorphic Ventricular Tachycardia. *Circ Res* 2018;123:953-63.10.1161/CIRCRESAHA.118.313369.

3. Magoc T, Salzberg SL. FLASH: fast length adjustment of short reads to improve genome assemblies. *Bioinformatics* 2011;27:2957-63.10.1093/bioinformatics/btr507.

4. Shen W, Le S, Li Y, Hu F. SeqKit: A Cross-Platform and Ultrafast Toolkit for FASTA/Q File Manipulation. *PLoS One* 2016;11:e0163962.10.1371/journal.pone.0163962.

5. Clement K, Rees H, Canver MC, et al. CRISPResso2 provides accurate and rapid genome editing sequence analysis. *Nat Biotechnol* 2019;37:224-6.10.1038/s41587-019-0032-3.

6. Li N, Wehrens XH. Programmed electrical stimulation in mice. *J Vis Exp* 201010.3791/1730.10.3791/1730.

7. Quick AP, Wang Q, Philippen LE, et al. SPEG (Striated Muscle Preferentially Expressed Protein Kinase) Is Essential for Cardiac Function by Regulating Junctional Membrane Complex Activity. *Circ Res* 2017;120:110-9.10.1161/CIRCRESAHA.116.309977.

8. Campbell HM, Quick AP, Abu-Taha I, et al. Loss of SPEG Inhibitory Phosphorylation of Ryanodine Receptor Type-2 Promotes Atrial Fibrillation. *Circulation* 2020;142:1159-72.10.1161/CIRCULATIONAHA.120.045791.

9. Picht E, Zima AV, Blatter LA, Bers DM. SparkMaster: automated calcium spark analysis with ImageJ. *Am J Physiol Cell Physiol* 2007;293:C1073-81.10.1152/ajpcell.00586.2006.

10. Respress JL, van Oort RJ, Li N, et al. Role of RyR2 phosphorylation at S2814 during heart failure progression. *Circ Res* 2012;110:1474-83.10.1161/CIRCRESAHA.112.268094.
